# Differential protein repertoires related to sperm function identified in extracellular vesicles (EVs) in seminal plasma of distinct fertility buffalo (*Bubalus bubalis*) bulls

**DOI:** 10.3389/fcell.2024.1400323

**Published:** 2024-07-29

**Authors:** Shiva Badrhan, Seema Karanwal, Ankit Pal, Jatinder Singh Chera, Vitika Chauhan, Aditya Patel, Mukesh Bhakat, Tirtha K. Datta, Rakesh Kumar

**Affiliations:** ^1^ Animal Genomics Laboratory, Animal Biotechnology Division, National Dairy Research Institute, Karnal, India; ^2^ ICAR- Central Institute of Research on Goat, Mathura, Uttar Pradesh, India; ^3^ Central Institute for Research on Buffaloes, Hisar, Haryana, India

**Keywords:** buffalo, bull fertility, seminal plasma extracellular vesicles, high-throughput proteomics, immunolocalization

## Abstract

Buffalo bulls are backbone of Indian dairy industry, and the quality of semen donating bulls determine the overall production efficiency of dairy farms. Seminal plasma harbor millions of lipid bilayer nanovesicles known as extracellular vesicles (EVs). These EVs carry a heterogenous cargo of essential biomolecules including fertility-associated proteins which contribute to fertilizing potential of spermatozoa. In this study, we explored size, concentration, and complete proteome profiles of SP EVs from two distinct fertility groups to uncover proteins influencing bull fertility. Through Dynamic Light Scattering (DLS) it was found that purified EVs were present in 7–14 size exclusion chromatographic (SEC) fractions with sizes ranging from 146.5 to 258.7 nm in high fertile (HF) and low fertile (LF) bulls. Nanoparticle Tracking Analysis (NTA) confirmed the size of seminal EVs up to 200 nm, and concentrations varying from 2.84 to 6.82 × 10^11^ and 3.57 to 7.74 × 10^11^ particles per ml in HF and LF bulls, respectively. No significant difference was observed in size and concentration of seminal EVs between two groups. We identified a total of 1,862 and 1,807 proteins in seminal EVs of HF and LF bulls, respectively using high throughput LC-MS/MS approach. Out of these total proteins, 1,754 proteins were common in both groups and about 87 proteins were highly abundant in HF group while 1,292 were less abundant as compared to LF bulls. Gene ontology (GO) analysis, revealed that highly abundant proteins in HF group were mainly part of the nucleus and involved in nucleosome assembly along with DNA binding. Additionally, highly abundant proteins in EVs of HF group were found to be involved in spermatogenesis, motility, acrosome reaction, capacitation, gamete fusion, and cryotolerance. Two highly abundant proteins, protein disulfide-isomerase A4 and gelsolin, are associated with sperm-oocyte fusion and acrosome reaction, respectively, and their immunolocalization on spermatozoa may indicate that these proteins are transferred through EVs. Our evidences support that proteins in EVs and subsequently their presence on sperm, are strongly associated with sperm functions. Altogether, our investigation indicates that SPEVs possess crucial protein repertoires that are essential for enhancing sperm fertilizing capacity.

## 1 Introduction

Spermatozoa are exquisitely crafted, motile gametes possessing the male’s genetic information which is transferred to the next-generation upon fertilization. In mammals, spermatozoa are suspended in seminal plasma (SP), which is a complex fluid consisting of bioactive molecules produced by secretions from the male reproductive tract, mainly from the epididymis and accessory sex glands (Juyena and Stelletta, 2012). SP accompanies spermatozoa from its maturation up to the time of its deposition in the female reproductive tract (FRT). It directly influences the sperms’ essential functions such as motility, capacitation, protection from oxidative stress, and modulation of the uterine immune response (Potts et al., 2000; Boccia et al., 2013; Rodríguez et al., 2013; Pereira et al., 2017). In addition to free bioactive molecules, some biomolecules including proteins, lipids, and nucleic acids in SP are encased in 30–350 nm lipid bilayer extracellular vesicles (EVs) which are delivered to the various target cells and hence, are essential for cell-to-cell communication (Zaborowski et al., 2015; Keerthikumar et al., 2016; Jeppesen et al., 2019).

Spermatozoa are hypothesized to be transcriptionally and translationally inactive and as a result, they carry pre-synthesized proteins as final products (Rahman et al., 2013; Sun et al., 2021). Consequently, EVs play a pivotal role in delivering the necessary protein cargo to meet the requirements of spermatozoa (Leahy et al., 2020). Most seminal EVs are secreted by the epididymis and prostate gland which are referred to as epididymosomes and prostasomes, respectively (Aalberts et al., 2012; Barrachina et al., 2022). Epididymosomes show different proteome profiles along the length of epididymis which lead to the maturation of spermatozoa, acquisition of motility, and the ability to bind with zona pellucida (Girouard et al., 2011; Rowlison et al., 2020). The ZP family proteins ZP3R and ZPBP2 in mouse epididymosomes have been shown to play a crucial role in zona-pellucida binding (Nixon et al., 2019). Seminal EV proteins in sheep have been found to correlate with vesicle biogenesis, metabolism, and membrane adhesion proteins which are essential for sperm fertilizing capacity (Leahy et al., 2020). In a study of seminal EVs from roosters of contrasting fertility, smaller EVs appeared to be more abundant in high fertile when compared to sub-fertile roosters (Cordeiro et al., 2021).

Seminal EVs are also known to enhance essential spermatozoal functions. In a previous report, exosomes from normal spermatozoa when co-incubated with spermatozoa from astenozoospermic men were able to enhance their progressive motility, capacitation, and acrosome reaction (Murdica et al., 2019). EVs with different densities are also known to differentially improve progressive motility and capacitation, among which the EVs with the highest density exhibited maximum improvement while the medium-density EVs were found to be enriched with antioxidant enzymes like GSTM2 (Wang et al., 2022). In a study on porcine semen, seminal EVs promoted acrosome reaction when they were co-incubated with spermatozoa (Siciliano et al., 2008). Prostasomes have also been reported to respond to progesterone and deliver Ca^2+^ under capacitating conditions (Aalberts et al., 2013). Additionally, EVs interact with endometrial stromal cells, promoting decidualization and prolactin secretion for implantation (Rodriguez-Caro et al., 2019). Additionally, seminal EVs from high fertile (HF) and low fertile (LF) animals display key differences in the type of cargo they carry. For example, cysteine-rich secretory protein 1 (CRISP1) was significantly less abundant in exosomes from astenozoospermic samples (Murdica et al., 2019). CRISP1 has been reported to regulate CatSper, the principal calcium channel in sperm that modulates sperm orientation and hyperactivation (Ernesto et al., 2015). Another protein Clusterin that plays a key role in capacitation and motility is less abundant in seminal EVs of non-normozoospermic men (Vickram et al., 2022). In another study, epididymal EVs from Teratospermic cats showed a significant deficiency in zona pellucida binding protein 1 which helps in species-specific sperm binding with oocyte when compared to normozoospermic cats (Rowlison et al., 2020). The sEVs not only transfer proteins but are also major carriers of miRNA, mRNA, piRNA, and snRNA. In a comprehensive study focusing on boar seminal EVs, the analysis revealed the presence of 288 known miRNAs, 37 novel miRNAs, and 19,749 piRNAs. Some of these mRNAs are known to modulate male reproductive physiology (Xu et al., 2020). Despite being one of the bodily fluids in which EVs were first identified nearly 50 years ago (Metz et al., 1968), SP has received less research focus in the context of EVs when compared to other bodily fluids (Royo et al., 2020). Research on the proteomic content of seminal extracellular vesicles (EVs) and its connection to buffalo bull fertility is a relatively unexplored field. To bridge this gap using a high throughput LC-MS/MS approach, the current study aims to identify differentially abundant proteins in the seminal EVs of Murrah buffalo bulls with distinct fertility and to elucidate their potential roles in fertilizing capacity.

## 2 Materials and methods

### 2.1 Chemicals and plastic wares

All chemicals and reagents used in this study were obtained from Sigma-Aldrich, India, ThermoFisher Scientific, and Invitrogen, United States, unless stated otherwise. Plasticwares were procured from Tarsons, India, and ThermoFisher Scientific, United States.

### 2.2 Selection of bulls and semen collection

Murrah bulls (*Bubalus bubalis*) were selected based on their conception rate (CR) data obtained from ABRC, NDRI, and the Central Institute for Research on Buffaloes (CIRB), Hisar. The buffalo bulls (n = 6) used in the study were categorized into two groups: high-fertile (HF, n = 3) with CR ranging from 38% to 48%, and low-fertile (LF, n = 3) with CR ranging from 6% to 34% (Supplementary Table S1) (Verma et al., 2014; Batra et al., 2020; Karanwal et al., 2023). All the bulls were maintained in the Artificial Breeding Research Centre (ABRC), NDRI. The semen samples were collected as per the minimum standard for production of bovine frozen semen, at ABRC. The semen ejaculate for each bull was collected weekly and number of samples per animal collected were approximately 24. After semen collection the physical parameters of semen were determine such as volume, colour, sperm concentration using a coulter counter and total motility (% of the total sperm population both motile and nonmotile). Any ejaculates failing to meet minimum standards for production of bovine frozen semen were not included in the experimentation.

### 2.3 Isolation of seminal plasma

The collected semen ejaculates were transported to the laboratory at 37°C without any delay (within half an hour) and centrifuged at 1,520 g for 15 min. The supernatant was collected in another tube and was centrifuged at 850 g for 5 min at 37°C. The final supernatant obtained was used for isolation of EVs or kept at −80°C until further use.

### 2.4 Isolation of seminal EVs using size exclusion chromatography (SEC)

For size exclusion chromatography, the column packaging and EV isolation was performed as previously described with minimal changes (Böing et al., 2014). A 10 mL of Sepharose CL-2b (Sigma-Aldrich, CL2B300) was packed in 20 mL column and washed with PBS (pH- 7.4). For EV isolation, 1 mL SP (thawed/fresh) was loaded on the column with 1 mL elution buffer, and a total of 26 fractions of 0.5 mL each were collected and were named in numerical order. After elution, the column was washed with 1 SEC volume (1 SEC volume = 12 mL) of 0.5 M NaOH followed by 2X washing with 0.1% Triton X. The column was then washed with distilled water and 0.05% sodium azide (2x SEC volumes). The SEC column filled with sodium azide was kept at 4°C for later use.

### 2.5 Dynamic light scattering (DLS) analysis of EVs

The size distribution of EVs was measured by Dynamic light scattering using a Zetasizer Nano ZS ZEN3600 (Malvern Instruments, Malvern, United Kingdom). After the isolation of seminal EVs, EVs were analyzed by pooling two consecutive fractions (fraction 1–2, fraction 3–4, and so on). The DLS sample comprised of seminal EVs and PBS in a 1:1 ratio. To prevent aggregation, these samples were sonicated using an ultrasonic water bath for 2 min and the size of EVs was measured. Three independent measurements were performed for each sample and the result was the average of three readings. Data analysis was performed using Zetasizer software version 7.03.

### 2.6 Nanoparticle tracking analysis (NTA) of EVs

The concentration and size distribution measurements were carried out through NTA in Malvern NS300 system. To perform NTA, fractions 7–9, 10–12, and 13–15 were diluted with PBS in a ratio of 1:1,000 to reduce the number of valid tracks below 200 per image. Each sample was measured five times, and 30–60 s videos were collected with less than 200 valid tracks recorded per video, and then the particles tracked were calculated and plotted.

### 2.7 Transmission electron microscopy (TEM) of EVs

For TEM, the grid was prepared as described previously (Pal et al., 2023). For sample preparation, EVs from fraction 7–14 were pooled and concentrated to 500 µL and incubated for 2 h in 4% PFA. About 4 μL EV sample was loaded on glow-discharged Formvar film-coated 300-mesh transmission electron microscopy grids. The sample was air-dried for 15 min followed by 3X washing with PBS. The EVs were then fixed by incubating in 1% glutaraldehyde for 15 min. After fixation, the grid was washed thrice with MilliQ water and stained with 1% phosphotungstenic acid for 45 s. Grid imaging was performed using a JEM1400FLASH transmission electron microscope (JEOL USA Inc., Peabody, MA, United States) at 120 kV and viewed under ×250,000 magnification.

### 2.8 Characterization of EVs through Western blot

To isolate proteins, the 7–14 fractions were first concentrated from 4 mL to 300 µL. The concentrated EVs were then incubated with Radioimmunoprecipitation assay buffer (RIPA buffer, Sigma-Aldrich, United States), in a ratio of 1:1.25 and 2 µL protease inhibitor (ThermoFisher USA) for 1 h, followed by syringe sonication for 24 times. A 100 μL EV lysate was then added into 900 µL chilled acetone and incubated for 22 h at −80°C. After incubation, the lysate was then centrifuged at 16,000 to 17,000 g for 30 min at 4°C, and the protein was redissolved in 80 µL of 0.5 M Tris HCl (pH- 6.8). For quantification of the protein, a standard Bradford graph was prepared using “Quick Start Bradford assay kit” according to the manufacturer’s instructions. SDS-PAGE of the protein was performed to evaluate its quality by utilizing the protocol described earlier (Karanwal et al., 2023).

To detect the EV biomarkers TSG101 and CD63, a Western blot assay was performed as described earlier (Pal et al., 2023). In brief, protein from HF and LF was separately pooled, then electrophoresed through SDS-PAGE was transferred from the gel to Immobilon-FL polyvinylidene difluoride membranes (PVDF, Millipore, Billerica, MA, United States). Before the transfer, membrane was first charged by immersing it for 1 min each in methanol and water. Subsequently, the membrane and the gel were immersed in the transfer buffer. The proteins on the gel were transferred onto PVDF membrane by using a power blotter station semi-dry blot (Invitrogen, United States) using voltage and current of 25 V and 2.5 A respectively at RT for 20 min. The blot was blocked by 5% BSA in Tris buffer saline containing Tween-20 (TBS-T) and incubated overnight at 4°C with gentle agitation. The membrane was incubated with primary mouse polyclonal anti-TSG101 (dilution-1:10,000, SC-7964, Santa Cruz Biotechnology, United States) and anti-CD63 (dilution-1:30,000, STJI40029, St. John’s laboratory, London, United Kingdom). Subsequently, the membrane was washed 3X with TBS-T for 15 min. The membrane was then incubated with HRP-conjugated secondary antibodies such as an anti-mouse secondary antibody for TSG101 (dilution-1:25,000, SC-516102, Santa Cruz Biotechnology) and an anti-rabbit secondary antibody for CD63 (dilution–1:140,000, STJ99512, St. Johns laboratory) for 2 h with constant shaking. The membrane was then washed 5X with TBS-T and then the immunocomplexes were detected using the ECL-HRP substrate (WBULS0100, Sigma). Finally, the signal was captured on an X-ray film and its image was taken using an Epson-perfection v600 film scanner, United States.

### 2.9 Preparation of protein samples for LC-MS/MS

For LC-MS/MS sample preparation, 50 µg seminal EV protein from each biological replicate was pooled to produce 150 µg protein from each fertility group. Out of 150 µg stock solution, 50 µg of protein sample was digested and reduced with 5 mM tris(2-carboxyethyl) phosphine (TCEP). The protein sample was further alkylated with 50 mM iodoacetamide and digested with trypsin (1:50, Trypsin/lysate ratio), and then incubated for 16 h at 37°C. The dried pellet was resuspended in buffer A (2% acetonitrile, 0.1% formic acid) for downstream LC-MS/MS analysis.

### 2.10 LC-MS/MS analysis of peptide mixtures and data processing

LC-MS/MS analysis was performed by utilizing an Easy-nlc-1000 system coupled with an Orbitrap Exploris mass spectrometer (Thermo Fisher Scientific, United States). To generate peptide spectra, 1 µg of peptide sample was injected in C18 column 15 cm, 3.0 μm Acclaim PepMap and separated with a 0%–40% gradient of buffer B (80% acetonitrile, 0.1% formic acid) at a flow rate of 300 μL/min. MS1 spectra were acquired in the Orbitrap (Max IT = 25 ms, AGQ target = 300%; RF Lens = 70%; R = 60K, mass range = 375–1,500).

The raw data from six technical replicates, three each for HF and LF groups were processed by retrieving the raw files. The data was analyzed with Proteome Discoverer (v2.5) against the UniProt Bovine database. Briefly, the precursor and fragment mass tolerances were set at 10 ppm and 0.02 Da respectively for Sequest and Amanda search. Both peptide spectrum match and false discovery rate (FDR) was set to <0.01 FDR to increase the confidence and remove the false positives. Proteins with Unique peptide ≥1 were selected and Keratin proteins were removed. Proteins with log2 fold change ≥2 were considered high abundant in HF and proteins with log2 fold change ≤ 0.5 were considered low abundant in HF bulls. Further, these proteins were divided into four categories namely, Known, predicted, uncharacterized, and LOC (i.e., when a published reference is not available and orthologs have not yet been determined, gene will provide a symbol that is known as “LOC” + gene ID) (Karanwal et al., 2023). The composition of unique proteins in HF and LF, and common proteins between HF and LF were visualized through Venn diagrams that were created using an online tool (https://bioinformatics.psb.ugent.be/webtools/Venn/).

### 2.11 Gene ontology and KEGG pathway analysis

To understand the functional roles of the identified proteins, differentially abundant proteins (DAPs) and unique proteins from both fertility groups were subjected to GO (Gene Ontology) analysis and KEGG (Kyoto Encyclopedia of Gene and Genomes) pathway analysis. The functional profiling of DAPs and unique proteins was carried out using UniProt and the Database for Annotation, Visualization, and Integrated Discovery (DAVID) gene enrichment tool v6.8. The proteins were categorized based on GO terms including cellular component (CC), molecular function (MF), and biological function (BP). Further KEGG pathway analysis was executed to understand the functional roles of these shortlisted proteins and their involvement in different pathways.

### 2.12 Immuno-localization of selected proteins on spermatozoa

To immunolocalize the selected proteins on spermatozoa, 100 µL of fresh semen was washed by adding 8 mL of sp-TALP (Sperm Tyrode’s albumin lactate pyruvate −50 mM NaCl, 5 mM HEPES, 1.55 mM KCl, 0.2 mM EDTA, 0.2 mM MgCl_2_.6H_2_O, 0.15 mM NaH_2_PO_4_.2H_2_O, 1 mM 60% Na-lactate, and 0.98 Mm Na-Pyruvate) followed by centrifugation at 800 g at 37°C for 7 min followed by incubation for 15 min in the incubator (New Brunswick, United Kingdom) with 5% CO_2_ maintained at 37°C. The washing step was repeated thrice. About 10 µL spermatozoa pellet was added on top of a poly-L lysine-coated glass slide (Poly-prep slides, Sigma-Aldrich) and were fixed using 4% Paraformaldehyde. Thereafter, the slide was washed thrice in 1X PBS for 2 min each. After washing, the slides were incubated in a permeabilization solution for 15 min (0.1% Triton X-100 in PBS) followed by three washing in PBS for 2 min each. Blocking was done for 1 h in 3% BSA prepared in PBST (0.1% Tween 20 in 1X PBS). Subsequently, the slides were washed thrice in PBST followed by overnight incubation with primary antibodies namely, mouse anti-PDIA4 at a dilution of 1:500 (Catalog no - RPD774Mu01, Cloud-Clone Corp.) and mouse Monoclonal Anti-Gelsolin antibody (Catalog no - G4896, Sigma-Aldrich) at a dilution of 1:500. Thereafter, the slides were washed 5 times in PBST. FITC labelled secondary goat anti-mouse IgG antibody (dilution 1:500, Catalog no–F4018, Sigma Aldrich) incubation was done for 1 h at RT. Following this, the slides were washed 5 times in PBST and counter-stained with 0.1 μg/mL DAPI (DNA stain Catalog no. D9542, Sigma Aldrich) for 1 min. The stained slides were washed 5 times in PBST for 2 min each. Thereafter, the slides were dried and a coverslip was mounted using DABCO to visualize the cells under the IX73 Olympus fluorescence microscope, United Kingdom at ×60 magnification. Along with immuno-localization of PDIA4 and GSN, secondary antibody control experiment was also performed (dilution 1:500) (Supplementary Figure S3).

## 3 Results

### 3.1 Isolation and characterization of seminal EVs

**FIGURE 1:**
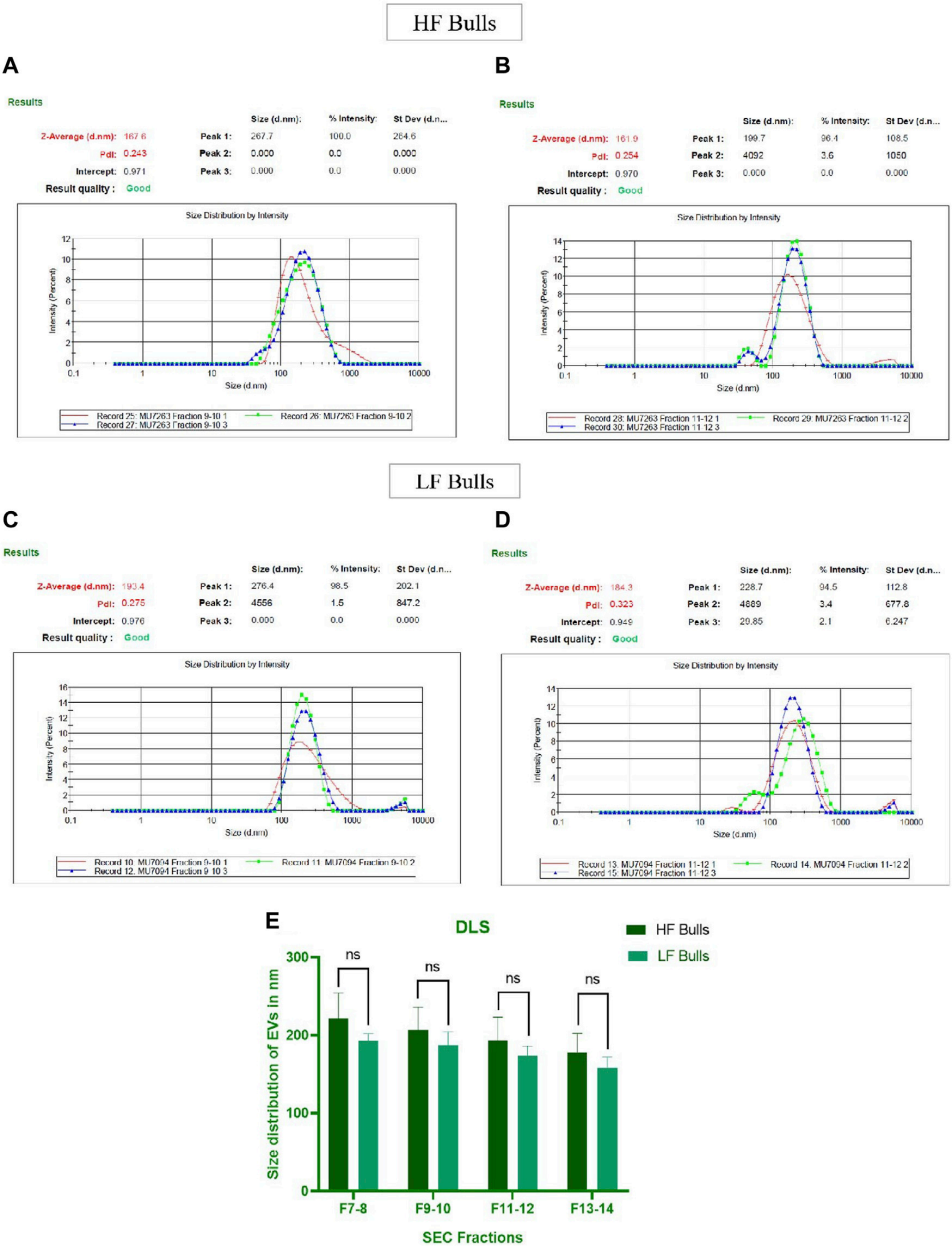
Characterization of seminal EVs. Intensity-based size distribution of seminal EVs analyzed by Zetasizer nano zs particle sizer. Each graph shows mean ± SD (n = 3), PDI, and particle size distribution for **(A)** Fractions 9–10 **(B)** fractions 11–12 of seminal EVs of HF bulls, and **(C)** fractions 9–10 **(D)** fractions 11–12 of seminal EVs from LF bulls. **(E)** Comparison of seminal EV size between the HF and LF bulls in which the *x*-axis represents the SEC fractions, and the y-axis represents the size of EVs in “nm.” The obtained *p*-value (0.1161) indicated that there was no statistically significant difference between the size of seminal EVs.

**FIGURE 2:**
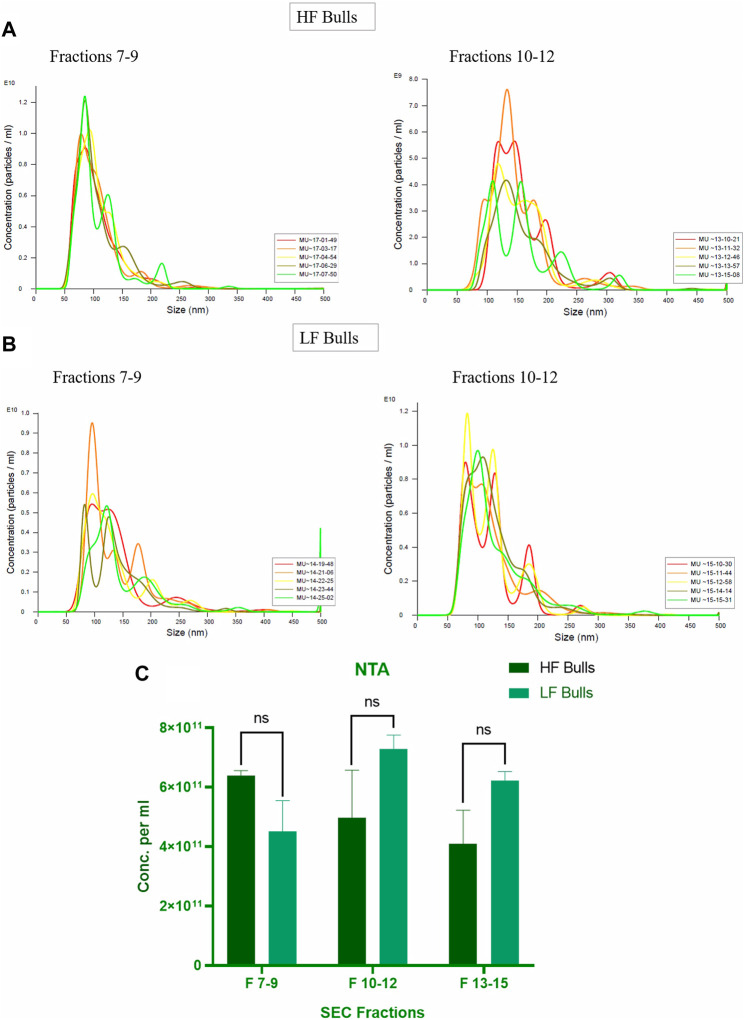
Characterization of seminal EVs Through Nanoparticle Tracking Analysis (NTA). The EVs were diluted 100X for NTA. The concentration vs. size graphs were plotted for **(A)** fractions 7–9, 10–12 of HF bulls and **(B)** for fractions 7–9, 10–12 of LF bulls. **(C)** Comparison of seminal EV concentration between the HF and LF bulls in which the x-axis represents the SEC fractions, and the y-axis represents the concentration of EVs in “particles per mL.” The obtained *p*-value (0.4588) indicated that there was no significant difference between the concentration of the seminal EVs between the HF and the LF groups.

**FIGURE 3:**
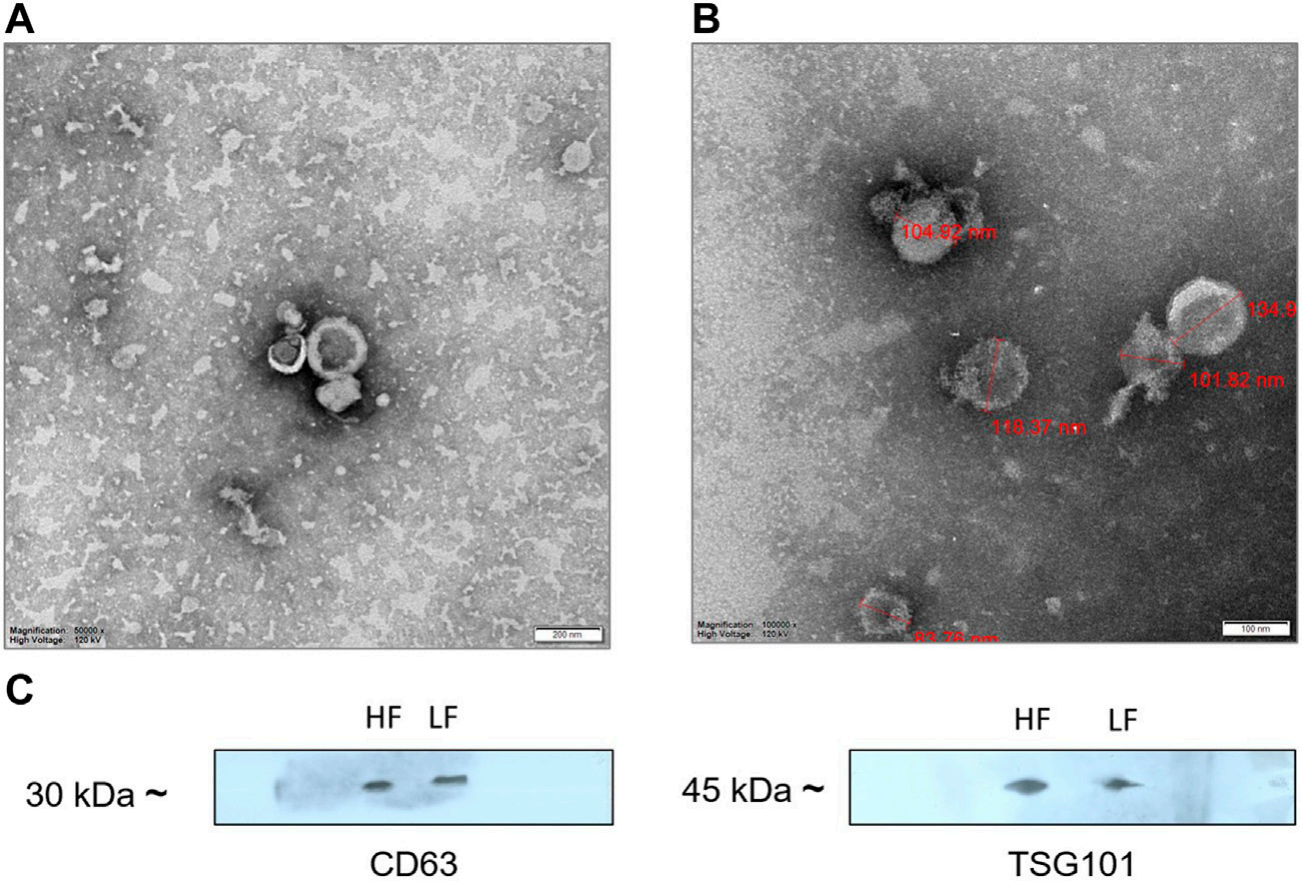
TEM images of **(A)** HF bulls **(B)** LF bulls seminal EVs that were negatively stained with phosphotungstic acid stain captured at ×250,000 magnification; scale bar–200 nm and 100 nm at 120 kV. **(C)** Identification of the CD63 and TSG101 EVs specific protein biomarkers by Western blot analysis. Protein extract loaded in each lane was 15 µg.

Seminal EVs were isolated using SEC, and all fractions were analyzed for the presence of seminal EVs using DLS. Out of 26 fractions, seminal EVs were mainly detected in SEC fractions of 7–8, 9–10, 11–12, and 13–14 as described earlier (Böing et al., 2014). Any differences in the concentration and the size of the EVs between the Murrah buffalo bulls of contrasting fertility were also assessed. The size of seminal EVs in the HF (high fertile) and LF (low fertile) bulls ranged from 146.5 nm to 200.3 nm and 160.1–258.7 nm, respectively (Supplementary Tables S2, S3). A *p*-value of 0.1161 indicated no significant difference in the size of the particles between the HF and the LF groups (Figure 1). The concentration of EVs was analyzed through NTA, and the results showed that in all the fractions (7–15), the concentration ranged from 2.84 × 10^11^ to 6.82 × 10^11^ particles per mL in the HF bulls, whereas in the case of LF bulls, the concentration of EVs ranged from 3.57 × 10^11^ to 7.74 × 10^11^ particles per mL (Supplementary Tables S4, S5). No significant difference was observed in the concentration of EVs between the HF and LF groups (*p*-value 0.4588) (Figure 2). Transmission electron microscopy (TEM) was employed to examine the morphology of SP EVs, which revealed their intact cup-shaped structure and membrane integrity in both, the HF and LF groups. The TEM results were consistent with both DLS and NTA findings and confirmed that the diameter of seminal EVs ranged from 50 to 150 nm (Figure 3). For biochemical characterization, the two EV biomarkers CD63 and TSG101 were confirmed for their presence in fractions 7–14 with molecular weight of 30 kDa and 45 kDa respectively (Supplementary Figure S2; Figure 3). The data collectively demonstrated the successful isolation of SP EVs by SEC.

### 3.2 Proteome profiling of seminal EV proteins of HF and LF bulls

**FIGURE 4:**
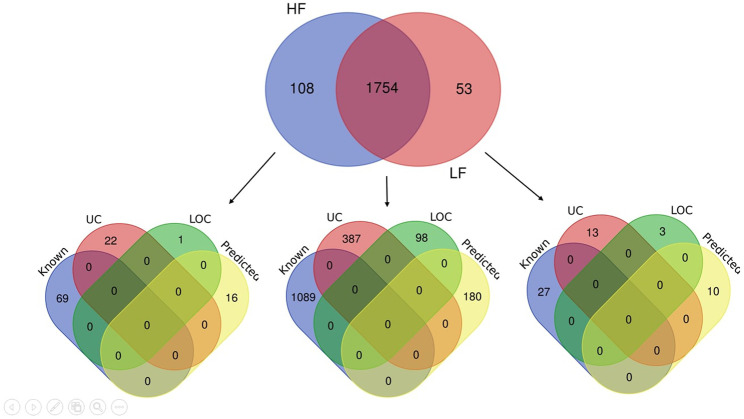
Venn diagram showing the number of identified proteins in seminal EVs of HF and LF bulls. A total of 1,862 and 1,807 proteins were present in seminal EVs of HF and LF bulls respectively. Out of these, 1,754 proteins were common (*p* < 0.05, FDR < 0.01). Some of the proteins were known in nature, followed by predicted, uncharacterized (UC), and LOC proteins.

**FIGURE 5:**
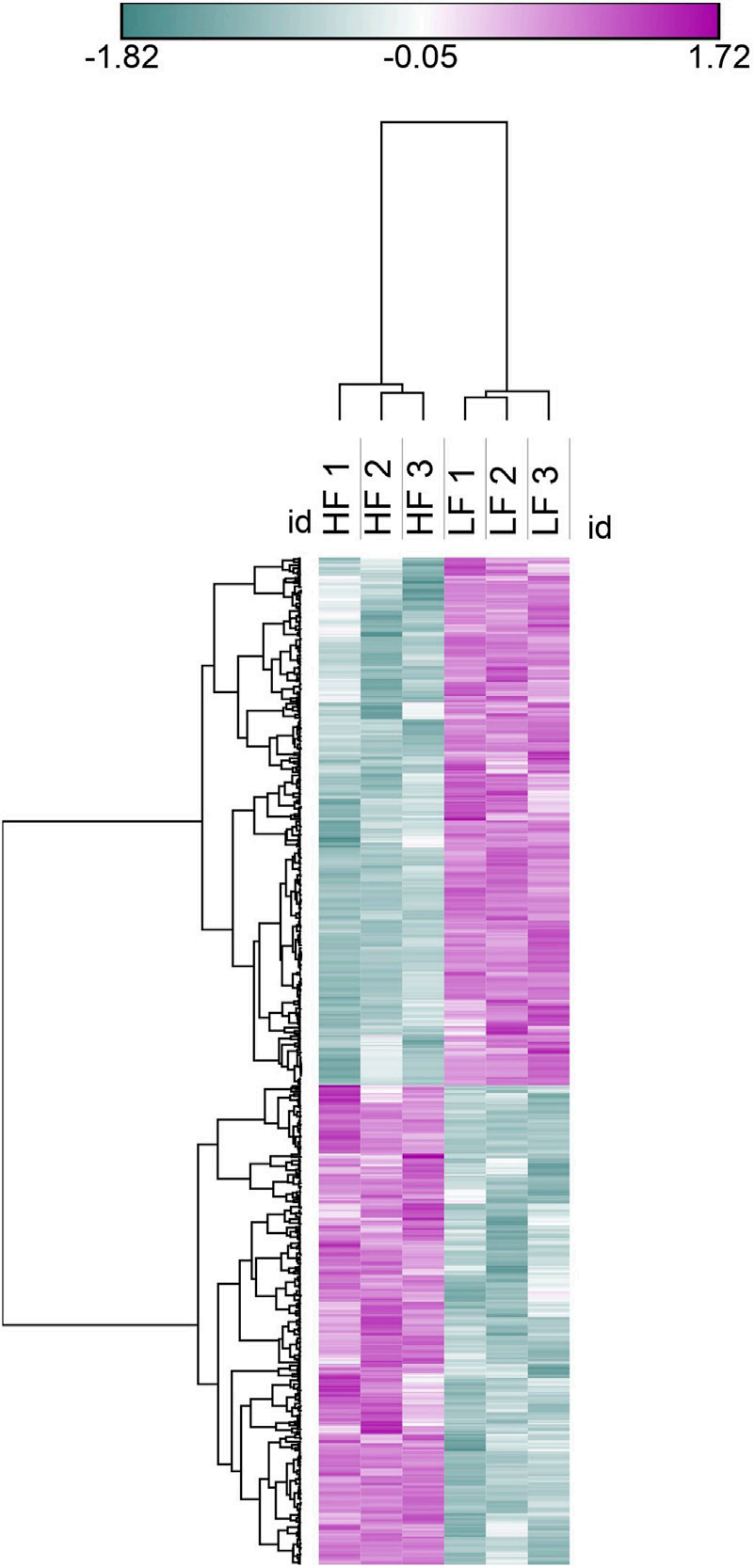
Heat map representation of DAPs in seminal EVs, Blue colour represents low abundant proteins in HF bulls and purple colour represents highly abundant proteins in HF bulls.

**FIGURE 6:**
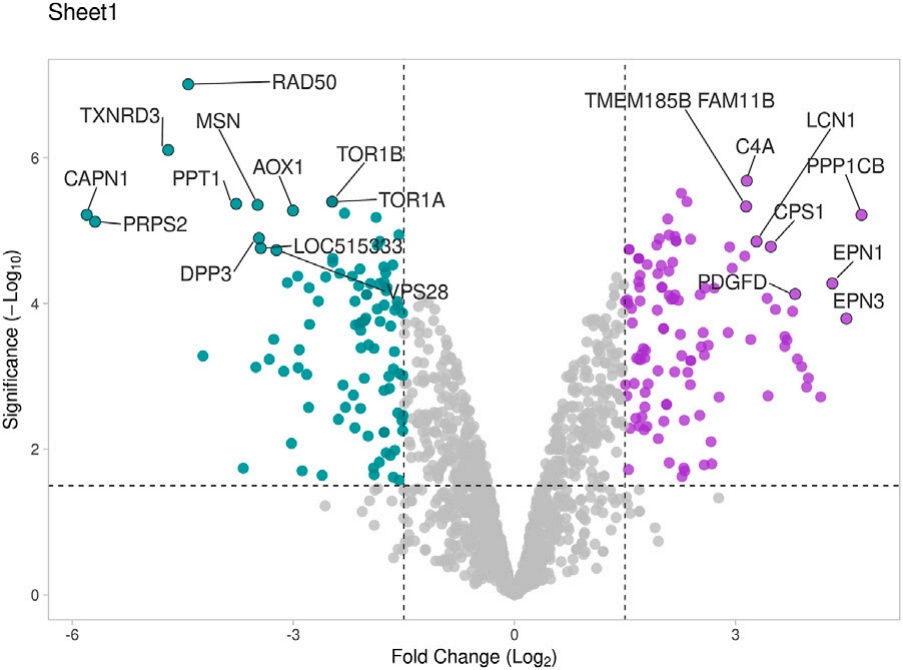
Volcano plot representing total DAPs identified in proteome profile of seminal EVs that were determined by log2 fold change vs. −log10 (*p*-value). Purple dots represent high abundant proteins in HF bulls, blue dots represent low abundant proteins in HF bulls, and grey dots represent neutral abundance of proteins.

The seminal EV proteome profiling revealed that EVs from HF and LF bulls possessed 1862 and 1807 proteins, respectively. Out of the total proteins, 1,754 were common in both HF and LF bulls, while 108 and 53 proteins were unique to HF and LF bulls, respectively (FDR < 0.01, ≥1 peptide, *p* < 0.05). These common proteins were described as “Differentially Abundant Proteins” (DAPs). Among the DAPs, 87 proteins were highly abundant in HF bull EVs (log2 fold change ≥ 2), and 1,292 were low abundant in HF bull EVs (Log2 fold change ≤ 0.5) as compared to LF bull EVs. Further, these proteins were categorized into four groups: Known, Predicted, Characterized, and LOC. The number of HF unique, LF unique, and common proteins was represented in the Venn diagram (Figure 4). The DAPs were also visualized using a heatmap and volcano plot (Figures 5, 6).

### 3.3 GO and KEGG pathways enrichment of high abundant proteins in seminal EVs of HF bulls

**FIGURE 7:**
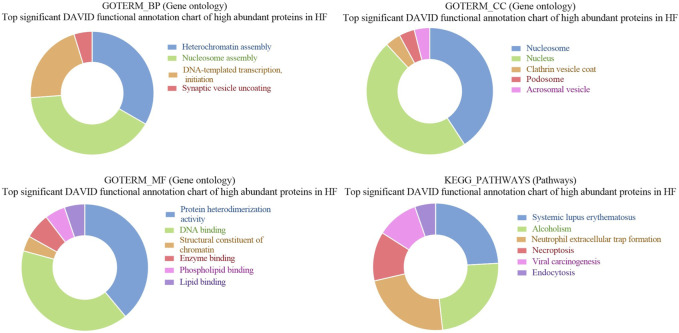
Pie chart representation of Gene ontology (GO) terms biological process (BP), Cellular component (CC), Molecular function (MF), and KEGG pathway enrichment of high abundant proteins in HF bulls.

GO of high abundant proteins in HF bulls revealed that these were associated with biological functions like nucleosome assembly (A7MAZ5, P62803, etc.; *p*-value = 1.3E-17), DNA templated transcription and initiation (P62803, G3X807, etc.; *p*-value = 9.4 E-13), and heterochromatin assembly (P0C0S9, F2Z4G5, etc.; *p*-value = 4.8E-20). In cellular components, many proteins were part of the nucleus (A7MAZ5, P0C0S9, etc.; *p*-value = 3.7E-6), nucleosome (F2Z416, Q3ZBX9, F2Z4J1, etc.; *p*-value = 1.3E-40), clathrin-coated vesicles (P04973, F1N579, and F1N4F8. *p*-value = 5.9E-4), and podosome (Q2KJA1, A4FUG5 and F1N1I6; *p*-value = 4.0E-3). In the case of molecular function, most proteins were involved in protein heteromerization activity (F2Z4I6, F2Z416, etc.; *p*-value = 5.9E-29) and DNA binding (F2Z4G6, F2Z4J1, etc.; *p*-value = 4.4E-19). KEGG pathway analysis revealed that the maximum number of proteins were involved in neutrophil extracellular trap formation (F1MLQ1, E1B7N2, G3N2B8, etc.; *p*-value = 4.4E-25) and necroptosis (P0C0S9, F2Z4G5, etc.; *p*-value = 4.7E-11) (Supplementary Table S6; Figure 7). Further, the list of high abundant proteins associated with fertility is given in Table 1 along with their log2 fold changes and *p*-values.

**TABLE 1:** List of top 20 fertility-associated proteins that were highly abundant in HF bulls along with their functions and log fold change.

S. no	Gene	Description	*p*-value	Log 2-fold change	Functions	References
1	PPP1C	Serine/threonine-protein phosphatase	0.0000433	5.67	It is essential for normal spermatogenesis	Sinha et al. (2013)
2	EPN1	Epsin 1	0.000053	4.31	It is a specialized endocytic adapter and its impairment leads to embryo lethality	Chen et al. (2009)
3	PDIA4	Protein disulfide-isomerase A4	0.001405066	3.96	It is highly expressed in boar semen and shows high cryotolerance	Tong et al. (2022)
4	PDGFD	Platelet-derived growth factor D	0.0000741	3.8	It regulates Proliferation and migration of gonocytes (PDGF-D act via PDGFR beta	Basciani et al. (2008)
5	NCKAP1	NCK associated protein 1	0.000128223	3.77	It regulates cytoskeleton organization during acrosome reaction	Tang et al. (2023)
6	ATP12A	Sodium/potassium-transporting ATPase subunit alpha	0.000388112	3.66	It is essential for sperm motility and fertility	Jimenez et al. (2011)
7	IZUMO1	Izumo sperm-egg fusion 1	0.000120773	3.54	Gamete fusion	Saito et al. (2019)
8	PPT1 CLN1 PPT	Palmitoyl-protein thioesterase 1 (Palmitoyl-protein hydrolase 1)	0.0000848	3.43	It affects sperm count, motility, and sperm morphology by regulating lysosomal function and cholesterol metabolism in Sertoli cells	Zhao et al. (2019)
9	ALCAM	Activated leukocyte cell adhesion molecule	0.000312308	3.2	It participates in the migration and cell-to-cell adhesion of Gonocytes with Sertoli cells	Ohbo et al. (2003)
10	TSSK2	Testis specific serine kinase 2	0.0000328	2.95	It has a role in Germ cell differentiation and sperm fertility	Salicioni et al. (2020)
11	CST3	Cystatin-C (Cystatin-3)	0.015864888	2.68	It is expressed in the FTR, binds to sperm, and prevents premature capacitation and AR—sperm motility	Lee et al. (2018)
12	TSSK1B TSSK1	Testis-specific serine/threonine-protein kinase 1	0.0000757	2.52	It has a role in Germ cell differentiation and sperm fertility	Salicioni et al. (2020)
13	—	Histone H4	0.0000107	2.41	Spermatogenesis, early embryo implantation, and sperm capacitation	Ugur et al. (2019)
14	LOC515333	ATP binding cassette subfamily C member 4	0.00000401	2.34	Reported to influence fertility traits	Neumann et al. (2023)
15	BHMT	Betaine homocysteine S-methyltransferase 1	0.004015387	2.3	It has role in epigenetic changes, spermatogenesis	Yuan et al. (2017)
16	ALDH2	Aldehyde dehydrogenase	0.01812099	2.29	It protects sperm from oxidative stress and promotes motility	Gibb et al. (2016)
17	PCSK4	Proprotein convertase subtilisin/kexin type 4	0.000265098	2.25	It plays important role in sperm fertilizing capacity, acrosome reaction, and capacitation	Iamsaard et al. (2011)
18	MDH2	Malate dehydrogenase	0.044397571	2.22	Regulation of energy in sperms	Pardede et al. (2020)
20	GSN	Gelsolin	0.019868739	2.31	Gelsolin helps in acrosome reaction by actin-depolymerizing. It is also involved in capacitation reaction	Finkelstein et al. (2010)
21	H2AC21	Histone H2A	0.001055687	3.98	Spermiogenesis	Wang et al. (2019)
22	PCSK4	Proprotein convertase subtilisin/kexin type 4	0.000265098	2.25	Sperm fertilizing capacity, acrosome reaction, and capacitation	Gyamera-Acheampong and Mbikay, (2009)
23	ZDHHC20	Palmitoyltransferase ZDHHC20	0.000520801	2.27	Gene expressed in testis and knock out of this gene in mice leads sperm to be unable to fertilize an egg	Wang et al. (2021)

### 3.4 GO and KEGG pathways enrichment of low abundant proteins in seminal EVs of HF bulls

**FIGURE 8:**
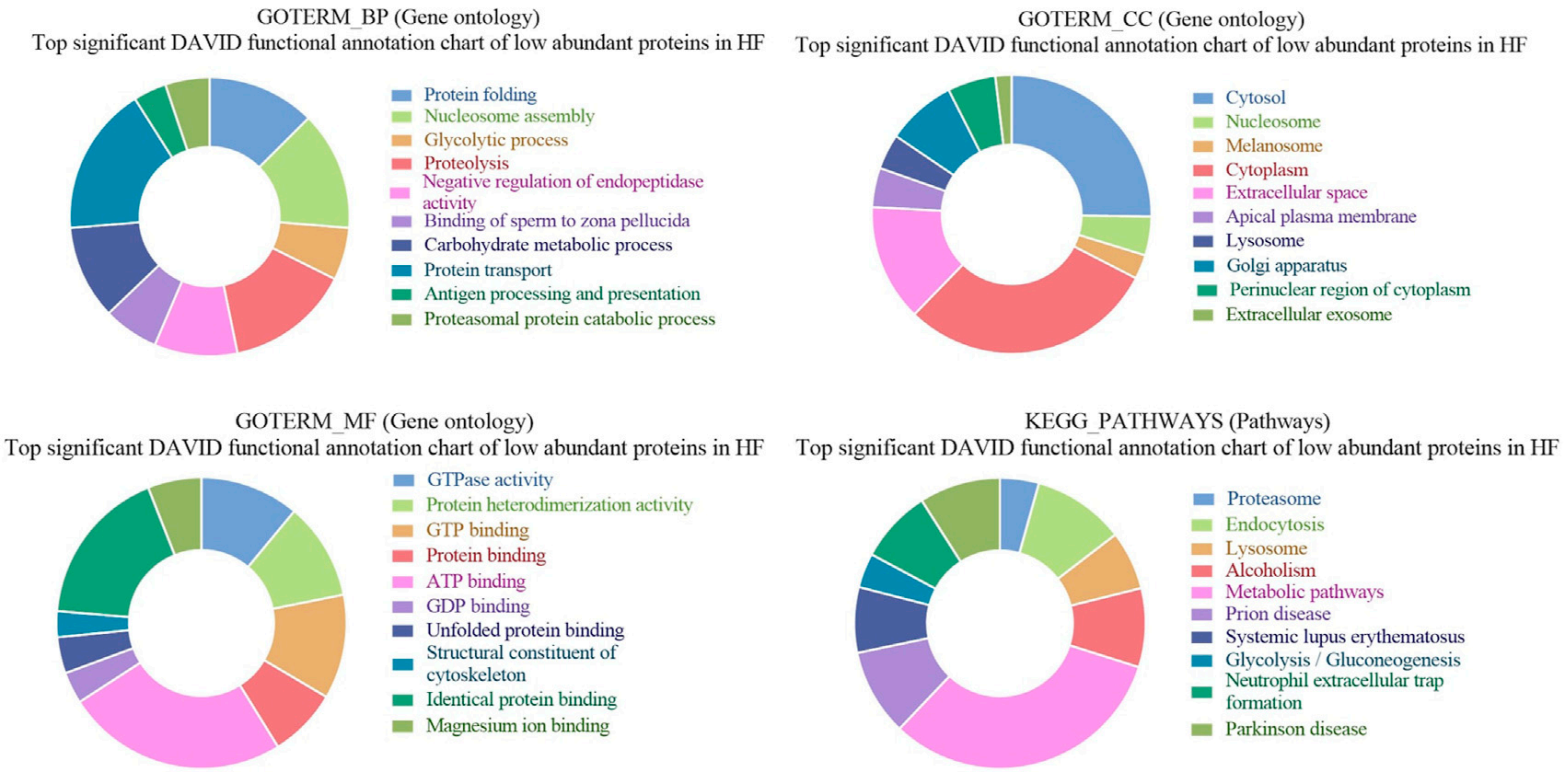
Pie chart representation of GO terms biological process (BP), Cellular component (CC), Molecular function (MF), and KEGG pathway enrichment of low abundant proteins in HF bulls.

Gene ontology analysis of low abundant proteins in HF bulls revealed that the majority of proteins were found to be associated with biological functions like protein transport (E1BP90, Q9XSK2, P2856, etc.; *p*-value = 1.5E-9), Glycolytic processes (E1B959, A6QLL8, F1MX69, etc.; *p*-value = 1.4E-12), proteolysis (Q3T0X8, Q5E946, G3NOF4, etc.; *p*-value = 1.9E-10), binding of sperm to zona pellucida (E1BG77, F1MFC4, Q32LB5, etc.; *p*-value = 7.3E-9). In cellular components, proteins were associated with cytosol (Q05927, Q0VCK0, P84080, etc.; *p*-value = 1.2E-36), Cytoplasm (F1MZP0, E1BDS9, P06623, etc.; *p*-value = 1.2E-34), apical plasma membrane (Q4GZT4, P31404, F1MXW4, etc.; *p*-value = 1.7E-22), melanosome (P06623, P31408, F1M132, etc.; *p*-value = 2.3E-22). In the case of molecular function, the proteins were associated with GTPase activity (Q5E196, G3N3N1, F1MN18, etc.; *p*-value = 2.4E-26), GTP binding (P84080, Q3SZF2, etc.; *p*-value = 3.4E-23), ATP binding (G3MXV4, E1BCR1, Q32PF2, etc.; *p*-value = 3.8E-19), protein binding (Q10741, P84080, etc.; *p*-value = 6.0E-16). In KEGG pathway analysis, the proteins were found to be associated with the proteasome (Q3T0X5, Q3T0Y5, Q3ZCK9, etc.; *p*-value = 1.9E-25), endocytosis (P884081, E1BP90, F1N371, etc.; *p*-value = 6.4E-21), metabolic pathways (F1MZP0, Q3ZCK3, Q05927, etc.; *p*-value = 9.8E-16) (Supplementary Table S6; Figure 8). The list of fertility-associated proteins that were low abundant in seminal EVs of HF bulls along with their function, Log2 fold change and the *p*-values is given in Table 2.

**TABLE 2:** List of fertility-associated proteins that were low abundant in HF bull EVs along with their functions and log fold changes.

S. no.	Gene	Description	*p*-value	Log 2-fold change	Function	References
1	CAPN1	Calpain-1 catalytic subunit	0.00000607	−5.8	This protein is associated with sperm motility	Cui et al. (2016)
2	PRPS2	Ribose-phosphate di-phosphokinase	0.00000755	−5.68	PRPS2 depletion contributes to the apoptosis of spermatogenic cells and is associated with hypo-spermatogenesis	Lei et al. (2020)
3	TXNRD3	Thioredoxin-disulfide reductase	0.000000782	−4.69	It plays a critical role via the thiol redox control of spermatogenesis	Dou et al. (2022)
4	RAD50	RAD50 double strand break repair protein	0.000000976	−4.425	Spermatogenesis	Hu et al. (2020)
5	CSE1L	Exportin-2 (chromosome segregation 1-like protein)	0.000524	−4.22	Spermatogenesis	Shi et al. (2022)
6	PPT1	Palmitoyl-protein thioesterase 1	0.0000043	−3.7749	Spermatogenesis	Zhao et al. (2019)
7	FSCN1	Fascin	0.0000518	−3.07	It is involved in sperm motility and morphology	Cheng et al. (2007)
8	SNF8	Vacuolar-sorting protein SNF8	0.008352	−3.02	It is an endocytosis gene upregulated in low-fertile bull sperm	Paul et al. (2021a)
9	AOX1	Aldehyde oxidase	0.00000531	−3.003	It is associated with decreased production of mature sperm	Saari et al. (2017)
10	CD81	CD81 antigen	0.003353	−2.97	It plays an important role in sperm-egg membrane fusion	Frolikova et al. (2018)
11	BOLA	MHC class I heavy chain	0.00076	−2.93	It helps in resistance to and appears to influence other traits such as milk yield, growth, and reproduction	Takeshima and Aida, (2006)
12	TSPAN8	Tetraspanin-8	0.000432	−2.91	It is a marker for spermatogonial subtype in immature mouse testis	Mutoji et al. (2016)
13	MUC15	Mucin-15	0.000944	−2.82	It is upregulated in low-quality semen	Karuthadurai et al. (2022)
14	CD151	CD151 antigen	0.0000606	−2.78	Acrosome reaction	Jankovicova et al. (2020)
15	RHOB	Rho-related GTP-binding protein RhoB	0.059873	−2.56	It regulates motility and the actin polymerization that accompanies the acrosome reaction	Castellano et al. (1997)

### 3.5 GO and KEGG pathways enrichment of unique proteins in seminal EVs of HF bulls

**FIGURE 9:**
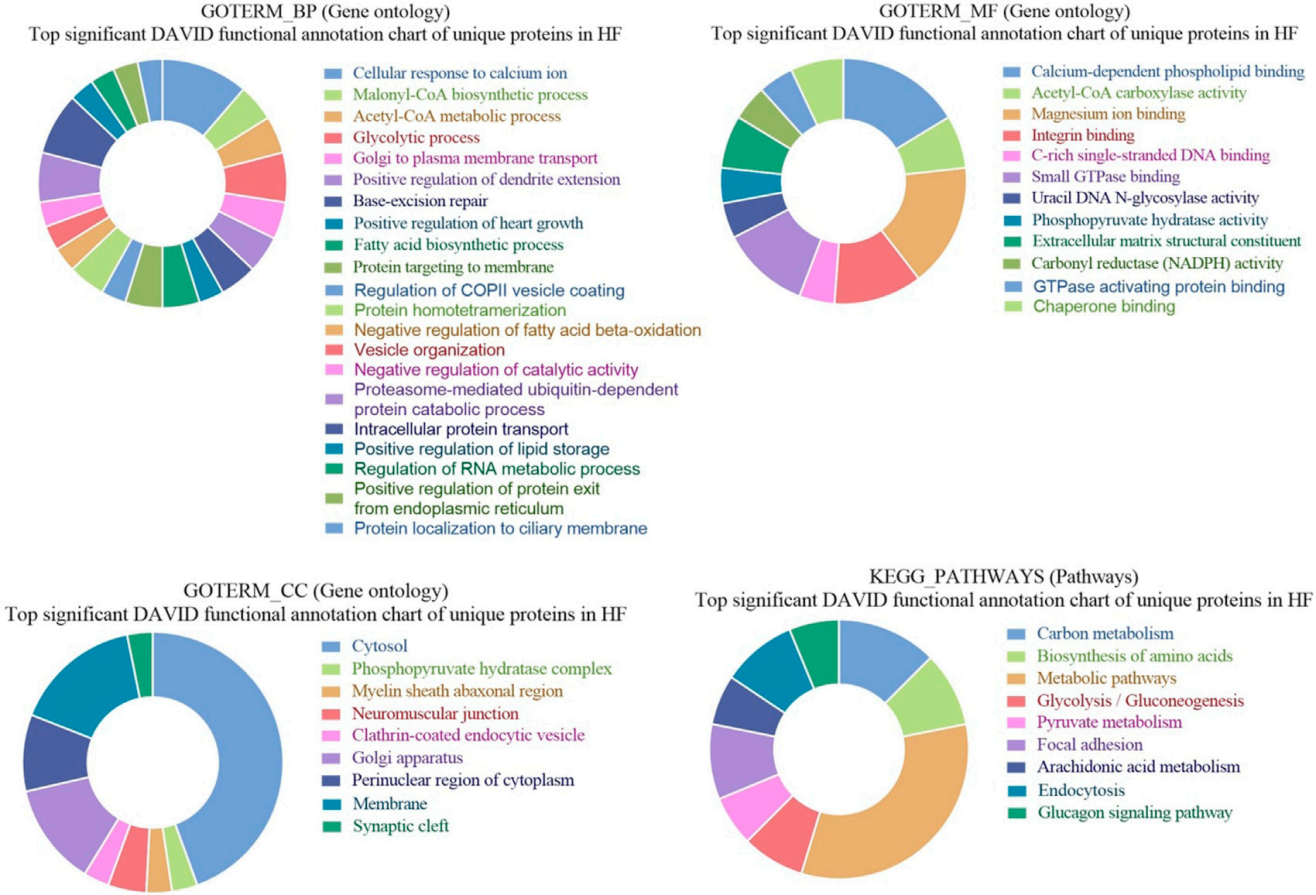
Pie chart representation of GO terms biological process (BP), Cellular component (CC), Molecular function (MF), and KEGG pathway enrichment of unique proteins in HF bulls.

GO of unique proteins in HF bulls revealed that these were associated with biological functions like a cellular response to calcium ion (A5D784, E1BID8, F1MLJ3; etc., *p*-value = 1.70E-06), malonyl-CoA biosynthetic process (E1BGH6, F1MSC3, Q9TTS3; *p*-value = 7.39E-05), acetyl-CoA metabolic process (E1BGH6, F1MSC3, Q9TTS3; *p*-value = 3.66E-04). In cellular components number of proteins were associated with organelles like cytosol (F6QS88, Q2KHU0, F1N189, etc.; *p*-value = 6.73E-05), phosphopyruvate hydratase complex (A6QR19, Q3ZC09; *p*-value = 0.016945), myelin sheath abaxonal region (A6QLD1, P53712; *p*-value = 0.021137), neuromuscular junction (Q2KJA7, F1MC13, P53712; *p*-value = 0.025453). In case of molecular function, many proteins were associated with GO terms like calcium-dependent phospholipid binding (A5D784, E1BID8, F1MLJ3, etc.; *p*-value = 1.71E-07), acetyl-CoA carboxylase activity (E1BGH6, F1MSC3, Q9TTS3; *p*-value = 5.80E-05), magnesium ion binding (F6QS88, G1K192, F1MWJ3, etc.; *p*-value = 2.98E-04), integrin binding (F1MDH3, Q2KJA7, F1MC13, etc.; *p*-value = 0.001399). In KEGG pathway analysis, the proteins were found to be associated with Carbon metabolism (Q148D3, A1A4J1, A6QR19, etc.; *p*-value = 2.13E-06), Biosynthesis of amino acids (A1A4J1, A6QR19, Q2KHU0, etc.; *p*-value = 4.39E-05), Metabolic pathways (F6QS88, Q3ZC09, Q2KHU0, etc.; *p*-value = 1.39E-04), Glycolysis/Gluconeogenesis (A1A4J1, A6QR19, Q3ZC09, etc.; *p*-value = 3.86E-04) (Figure 9).

### 3.6 GO and KEGG pathways enrichment of unique proteins in seminal EVs of LF bulls

**FIGURE 10:**
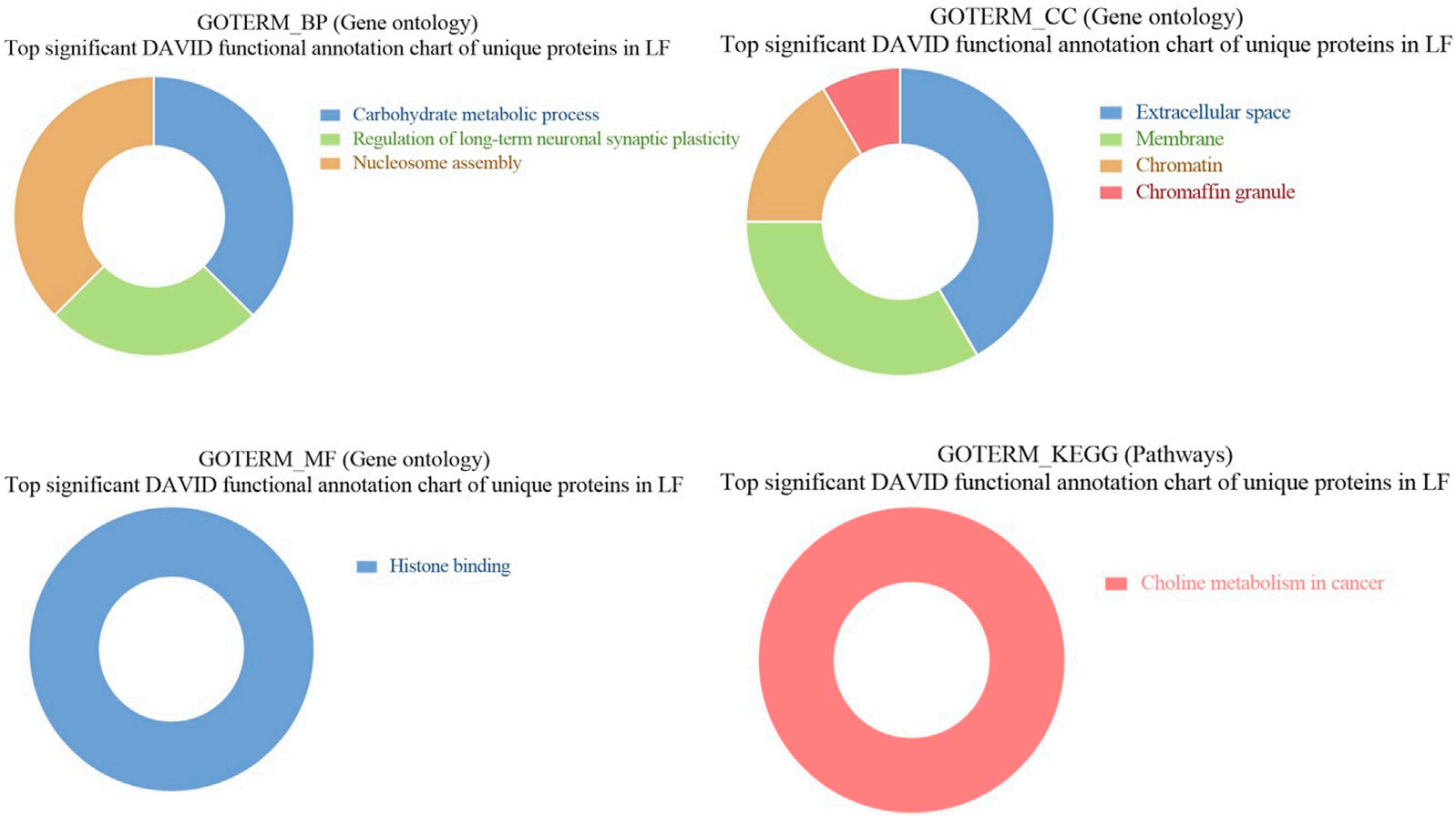
Pie chart representation of GO terms biological process (BP), Cellular component (CC), Molecular function (MF), and KEGG pathway enrichment of unique proteins in LF bulls.

GO of unique proteins in LF bulls revealed that these were associated with biological functions like carbohydrate metabolic process (Q5EAB4, G3MXK6, F1MDX6, A7MBC0; *p*-value = 0.027632), regulation of long-term neuronal synaptic (Q0IIG7, Q3SZ33; *p*-value = 0.029595), nucleosome assembly (A6H767, F1N7X3, Q2TA40, G3X7M5; *p*-value = 0.040927), negative regulation of intrinsic apoptotic signaling (G3MYD5, P68002; *p*-value = 0.058338). In cellular components, proteins were associated with extracellular space (Q8SPU5, Q5EAB4, E1B9Y3, etc.; 0.001648), membrane (F1N5M4, Q2TBQ5, P00171, etc.; *p*-value = 0.006135), chromatin (A6H767, F1N7X3, A6QLY7, etc.; *p*-value = 0.018316), chromaffin granule (F1MWI1, A6QPQ2; *p*-value = 0.026138). In the case of molecular function, the proteins were associated with histone binding (A6H767, F1N7X3, Q2TA40, G3X7M5; *p*-value = 0.031620912). In KEGG pathway analysis, the proteins were found to be associated with Choline metabolism in cancer (F1N5M4, Q2KJ15, A2VDK6; *p*-value = 0.009219) (Figure 10).

### 3.7 Immuno-localization of gelsolin and PDIA4 on the spermatozoa

**FIGURE 11:**
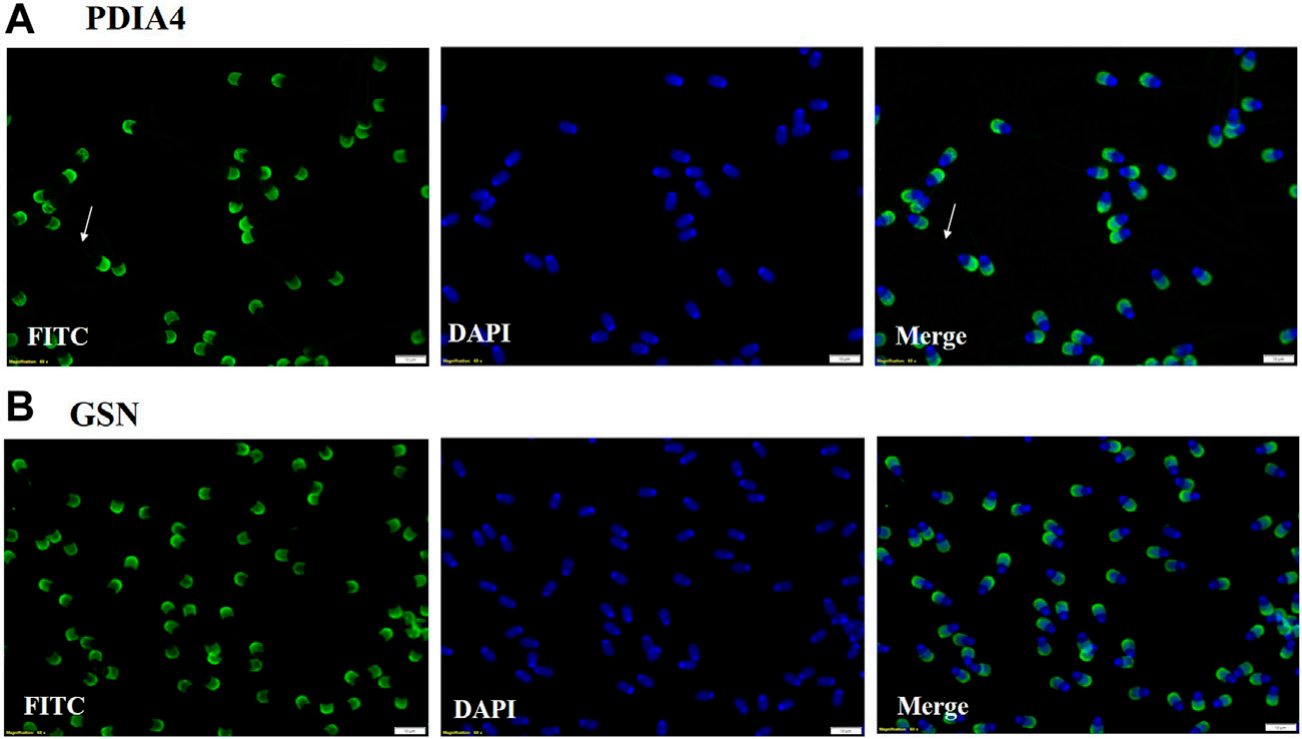
**(A)** Immuno-localization of PDIA4 was done on spermatozoa. ICC results show that PDIA4 is present on the acrosome region and mid-piece of spermatozoa. **(B)** Immuno-localization of Gelsolin on spermatozoa. ICC results showed that Gelsolin is present on the acrosome region of spermatozoa.

Two proteins PDIA4 and GSN that were highly abundant in HF bull EVs were subjected to immunolocalization on spermatozoa. It was observed that PDIA4 was present on the acrosome region and mid-piece of spermatozoa while Gelsolin was present on the acrosome region of the spermatozoa of bulls (Figure 11). In this study we also performed the secondary antibody control that does not shows any binding of secondary antibody on the spermatozoa (Supplementary Figure S3).

## 4 Discussion

This research was conducted to investigate whether there are differences in the size, concentration, and proteomic cargo of EVs based on the fertility status of buffalo bulls.

Seminal EVs were isolated using SEC and further characterized by DLS, NTA, TEM, and Western blotting. Fractions 7–14 carried the EVs with mean size of 200 nm in both the fertility groups as reported in previous studies (Luo et al., 2022; Barranco et al., 2023). The particles in the sample were homogenous as indicated by polydispersity index (PDI) ranging from 0.2 to 0.35 in the DLS data from EVs of both the fertility groups. In addition to the DLS method, the NTA assay re-established not only the size of the seminal EVs but also confirmed the abundance of EVs in buffalo SP. TEM also illustrated round cup-shaped characteristic morphology of seminal EVs in both HF and LF bulls falling in the desired size range (Xu et al., 2020; Yu et al., 2023).

The seminal EV proteomic profile of HF and LF bulls was deciphered with the help of LC-MS/MS and computational biology. From the data, 1,862 and 1,807 proteins were identified in HF and LF seminal EVs respectively. The highly abundant proteins present in HF bulls were further examined to determine their roles in the fertilizing potential of spermatozoa and based on previous literature, numerous proteins were found to be associated with the fertilizing capacity as mentioned in Tables 1, 2.

EVs are released by most internal organs of the male reproductive system like epithelial cells of vesicular glands (anatomically referred to as seminal vesicles) and the ampulla of the ductus deferens (Agrawal and Vanha-Perttula, 1987; Renneberg et al., 1995). Furthermore, Sertoli cells have also been reported to release EVs (Mancuso et al., 2018). All the EVs released by the organs constitute the diverse population of EVs present in SP.

Spermatogenesis is a complex series of events that includes the mitotic proliferation of spermatogonial cells, the meiotic division of spermatocytes, and their further maturation into spermatozoa. Various proteins involved in different aspects of sperm development were found to be highly abundant in HF seminal EVs. Activated leukocyte cell adhesion molecule (ALCAM) (log2 fold change - 3.2057; *p*-value = 0.00031) is expressed transiently in gonocytes and facilitates gonocyte-sertoli cell adhesion and migration of gonoyctes toward the basement membrane (Ohbo et al., 2003). Platelet-derived growth factor D (PDGFD) (log2 fold change - 3.8084; *p*-value = 7.41E-05) is localized in Sertoli cells in prenatal and early postnatal testis and its interaction with PDGFR-β is crucial for gonocyte development (Basciani et al., 2008). Testis-specific serine kinase 2 (TSSK 1,2) (TSSK1–log2 fold change-2.5187; *p*-value = 7.57E-05, TSSK2 – log2 fold-2.9543; *p*-value = 3.28E-05) are testis-specific kinases localized in spermatids and spermatozoa and deletion of *Tssk1* and *2* causes male infertility due to haploinsufficiency (Xu et al., 2008), TSSKs have a crucial role in germ cell differentiation and sperm function (Salicioni et al., 2020). Betaine--homocysteine S-methyltransferase 1 (BHMT) (log2 fold change - 2.3045; *p*-value = 0.00401) is an important methyl donor and aberrant expression of BHMT leads to global DNA hypomethylation and modulation of spermiogenesis (Zhang et al., 2015; Yuan et al., 2017). Palmitoyl-protein thioesterase 1 (PPT1) (log2 fold change–3.4280; *p*-value = 8.48E-05) is a lysosomal depalmitoylating enzyme and its absence in Sertoli cells leads to disruption in adhesion of developing germ cells to Sertoli cells that causes decline in motility and number of sperms along with increase in number of sperms with deformed heads (Zhao et al., 2019). Nudix hydrolase 5 (NUDT15) (log2 fold change = 2.3856; *p*-value = 0.00130) is a gene of the KRAB transcription family and it might be involved in early embryonic maintenance or methylation of genomic regions (Lalancette et al., 2009). Histone 4 was previously reported to be localized in head region of sperm in Holstein bulls. It showed high expression in HF bulls compared to LF (Ugur et al., 2019).

Motility is a substantial trait of spermatozoa and numerous proteins were found highly abundant in HF bulls that are known to play essential roles in motility. Protein phosphatase (log2 fold change = 2.5762; *p*-value = 0.000511) is predominantly expressed in the testis and distributed over the flagellum and post-acrosomal region of the sperm head in dog (Tash et al., 1988). PPP1CA, PPP1CB, and PPP1CC1 are ubiquitously present, and PPP1CC2 is present at high levels in adult testis and spermatozoa. Deletion of the Ppp1cc gene causes loss of both isoforms resulting in oligo-terato-asthenozoospermia and thus, causing male infertility due to impaired spermiogenesis (Sinha et al., 2013). Another protein Sodium/potassium-transporting ATPase subunit alpha (ATP12A) (log2 fold change = 3.6687; *p*-value = 0.00038) also showed high abundance in HF bulls EVs. Inhibition of ATP12A has been reported to cause impairment of sperm motility and capacitation *in vitro* (Escoffier et al., 2020). ATP12A is localized on the acrosome region of spermatozoa in *B. bubalis*, *Bos taurus*, and *Ovis aries* and its expression increases progressively in bovine sperm acrosome region while moving from epididymis to deferent ducts (Favia et al., 2022). Sodium/potassium-transporting ATPase subunit alpha has 3 isoforms out of which, alpha4 is expressed in male germ cells and localized on the flagellum of spermatozoa which plays a pivotal role in motility (Woo et al., 2000). Aldehyde dehydrogenase (ALDH2) (log2 fold change = 2.2975; *p*-value = 0.01812) was also highly abundant in HF bull EVs which also plays an essential role in sperm motility. A recent study on stallion sperm showed a positive correlation between ALDH expression and progressive as well as rapid motility. ALDH was expressed all over the spermatozoa and the strongest expression was observed in the head region and mid-piece (Gibb et al., 2016). It is also highly expressed in spermatozoa of HF bulls (Karanwal et al., 2023). Malate dehydrogenase MDH2 (log2 fold change = 2.2166; *p*-value = 0.04439) is associated with energy regulation of sperm (Pardede et al., 2020), and comparative proteomics in a previous report revealed MDH2 as a fertility biomarker in spermatozoa in crossbred bulls (Muhammad Aslam et al., 2018). MDH has also been found to be involved in the formation of acrosomal membrane in *Eriocheir sinensis* (Li et al., 2023).

Capacitation and acrosome reaction are two important processes that occur before the fusion of sperm and oocyte. Capacitation involves many orchestrated processes including activation of cAMP-dependent phosphorylation pathways, removal of cholesterol, hyperpolarization, and change in ion permeability of sperm plasma membrane (Stival et al., 2016). Proteomic analysis of seminal EVs revealed many proteins that have been reported to play crucial roles in capacitation and acrosome reaction. Cystatin-C (CST3) (log2 fold change = 2.6770; *p*-value = 0.01586) is highly expressed in the female reproductive tract and it can bind to the post-acrosomal head region, midpiece and tail of spermatozoa. It enhances sperm motility and inhibits premature capacitation (Lee et al., 2018). It is reported to be associated with prostasomes (Carlsson et al., 2011). Another protein Gelsolin (GSN) (log2 fold change = 2.3103; *p*-value = 0.01986) was also found to be highly abundant in HF bulls EVs. Gelsolin is inactive during capacitation and the release of bound gelsolin causes F-actin depolymerization and it also enhances acrosome reaction (Finkelstein et al., 2010). Proprotein convertase subtilisin/kexin type 4 (PCSK4) (log2 fold change = 2.2510; *p*-value = 0.00026) is essential in sperm fertilizing ability localized along the acrosomal plasma membrane of mammalian spermatozoa (Gyamera-Acheampong and Mbikay, 2009). Inhibition of PCSK4 in rat spermatozoa has been shown to interfere with oocyte penetration (Dahril et al., 2019). Izumo sperm-egg fusion 1 (IZUMO1) (log2 fold change = 3.5414; *p*-value = 0.00012), a sperm adhesion protein, was also found highly abundant in HF bull EVs which aids in sperm—egg fusion by interacting with oocyte-specific GPI-anchored receptor JUNO. The mouse lines with disrupted Izumo gene produced healthy mice with spermatozoa that penetrated zona pellucida but were unable to fuse with eggs (Saito et al., 2019). Palmitoyl transferase (ZDHHC20) (log2 fold = 2.2691; *p*-value = 0.00052) is a testis-enriched gene and it was also found to be highly abundant in HF bull sperm EVs. Its deletion results in an abnormality in sperm head and tail, decreased motility, and disturbed acrosome reaction leading to male sterility (Wang et al., 2021).

Approximately 26 percent of the detected DAPs were identified as histone proteins and modified histone proteins. These proteins are mostly comprised of H2A and H4 histones. In a recent report, H4 and acetylated H4 were more abundant in spermatozoa of HF bulls (Ugur et al., 2019). Histones and their modification play a significant role in DNA packaging (Miller et al., 2010). Previous studies demonstrated that H2B, H3.3, and H4 were detectable in bull spermatozoa, and the condensation status of sperm chromatin, influenced by histone retention, is associated with the fertility of bulls (De Oliveira et al., 2013). Two H2A variants H2AFV and H2AFZ that are known to influence global DNA methylation and spermatogenesis were also highly abundant in HF Murrah bull spermatozoa (Zhao et al., 2013; Madakashira et al., 2017; Karanwal et al., 2023).

In case of low-abundant proteins in HF bulls, three proteins were negatively correlated with semen quality. These proteins are Vacuolar-sorting protein SNF8, aldehyde oxidase, and Mucin 15. Vacuolar-sorting protein SNF8 (log2 fold = −3.2285; *p*-value = 1.85E-05), an endocytosis gene, was previously found to be upregulated in low-fertile bull sperm (Paul et al., 2021a), while aldehyde oxidase (AOX1) (log2 fold change = −3.0038; *p*-value = 5.31E-06) has been associated with decreased production of mature sperm (Saari et al., 2017). Additionally, Mucin 15 (MUC15) (log2 fold change = −2.8176; *p*-value = 0.00094) was found to be upregulated in low-quality semen (Karuthadurai et al., 2022). Along with our findings, the available reports substantiate that EVs not only carry proteins responsible for enhancing fertilizing potential but also those proteins that harm sperm quality. The data generated from this study show high abundance of these three proteins in LF bull seminal EVs, suggesting that they may collectively contribute to the low quality of semen in LF bulls.

Two of the significantly abundant proteins in HF seminal EVs were also immunolocalized on the spermatozoa. Protein disulfide isomerase A4 (PDIA4) (log2 fold change = 3.9638; *p*-value = 0.00140) is a protein localized in the endoplasmic reticulum (ER) lumen that catalyzes the formation, isomerization, and reduction of disulfide bonds (Turano et al., 2002). These PDI proteins are found in the equatorial segment of mature spermatozoa (Ellerman et al., 2006) that play a crucial role during sperm-oocyte fusion by forming multimolecular complexes that are critical for the fusion process (Yanagimachi, 1994). In the context of predicting the freezability of Erhualian boars, PDIA4 has been identified as a potential biomarker. Immunolocalization of PDIA4 on spermatozoa showed its presence on the acrosome region and mid-piece of spermatozoa. Another protein that was localized was Gelsolin (GSN) (log2 fold change = 2.3103; *p*-value = 0.01986) which plays an important role in the sperm acrosome reaction. The cortical cytoskeleton is disassembled during the exocytotic acrosome reaction, which fuses the plasma membrane with the outer cytoskeleton membrane. This barrier can be broken by proteins of the gelsolin family (Finkelstein et al., 2010). Gelsolin also showed high expression in spermatozoa of HF Murrah bulls (Karanwal et al., 2023). Immunolocalization of gelsolin on spermatozoa revealed that it is present in the acrosome region of spermatozoa. Our findings demonstrate that PDIA and gelsolin remained differentially abundant in seminal EVs and its detection on sperm indicates that these proteins might be getting transferred to the spermatozoa through EVs as spermatozoa are translationally inactive. However, further studies need to be carried out to robustly validate this claim. Therefore, the overall data collectively indicate that the fertility status of the Murrah bulls can be predicted based on the proteomic profile of their seminal EVs while their size and concentration do not exhibit significant differences with respect to fertility status. However, there are some limitations in the study. For example, the assessment of the field fertility in buffalo bulls faced limitations due to the small sample size, and evaluating their fertility takes a considerable amount of time. Samples from both groups were pooled for LC-MS/MS and Western blotting due to technical and financial constraints. However, the pooling of sample increases homogeneity within the group detecting a maximum number of proteins while maintaining a high degree of confidence (Park et al., 2012; Somashekar et al., 2017; Muhammad Aslam et al., 2019). Conducting additional experiments involving a larger sample size within the same group will undoubtedly contribute to a more comprehensive understanding of the findings presented in the current study. Additionally, Western blot experiments also need to be performed to establish the differential abundance of the seminal EV proteins that were detected through LC-MS/MS. Experiments involving coincubation of seminal EVs and spermatozoa will further aid in establishing the transfer of the protein cargo from EVs to spermatozoa along with their roles in enhancing or reducing sperm functions.

## 5 Conclusion

In conclusion, our study shows a discrete proteome profile of seminal EVs of high vis a vis low fertile buffalo bulls using label-free LC-MS/MS. In addition, our study also indicates that the fertility status of the buffalo bulls is not dependent on the size and concentration of the seminal plasma EVs. However, based on comparative proteome data of seminal EVs of two distinct fertility groups, we identified many differentially abundant proteins and further explained the most prominent proteins associated with sperm function. The highly abundant proteins identified in the seminal EVs of HF bulls were found to be associated with key sperm functions such as sperm motility, capacitation, acrosome reaction, and pellucida binding, reflecting their crucial and supportive roles in various sperm functions. LF bull EVs exhibited the presence of certain proteins with detrimental effects on semen quality, nevertheless, a few were associated with spermatogenesis and motility. The presence of PDIA4 and GSN on spermatozoa may indicate that these proteins are being transferred on the spermatozoa through EVs although, further studies are required to verify this claim. Altogether, this study provides a unique insight into the dynamics of protein repertoire in seminal EVs in relation to buffalo bull fertility. Our current findings need further validation using additional protein candidates and co-incubation experiments of EVs with spermatozoa and subsequently, monitoring the changes in the fertilizing potential of spermatozoa.

## Data Availability

The data presented in the study are deposited in the PRIDE repository, accession number; PXD050211.
