# Supplementary material for: Differential protein repertoires related to sperm function identified in extracellular vesicles (EVs) in seminal plasma of distinct fertility buffalo (Bubalus bubalis) bulls
Source: Front Cell Dev Biol. 2024 Jul 29;12:1400323. doi: 10.3389/fcell.2024.1400323 (PMC11318068; doi:10.3389/fcell.2024.1400323)
Supplement: Supplementary file 1 [file Table1.DOCX]

Supplementary Data

Supplementary data – Table S1: Selected bulls and their conception rates (CR).

| **Fertility groups** | **Buffalo Bulls ID no.** | **Total Al** | **Pregnancy** | **Conception Rate (%)** |
| --- | --- | --- | --- | --- |
| HF1 | MU7263 | 1429 | 682 | 47.73 |
| HF2 | MU7227 | 1724 | 819 | 47.51 |
| HF3 | MU7147 | 2155 | 821 | 38.1 |
| LF1 | MU7094 | 1719 | 581 | 33.8 |
| LF2 | MU7584 | 1041 | 181 | 17.39 |
| LF3 | MU7649 | 837 | 51 | 6.1 |

Supplementary data – Table S2 : Size distribution (nm) of fraction 7-14 in HF Bulls

| HF Bull No | F7-8 | F9-10 | F11-12 | F13-14 |
| --- | --- | --- | --- | --- |
| MU7263 | 181.4 | 167.6 | 161.9 | 146.5 |
| MU7227 | 199.7 | 193 | 176.1 | 154.7 |
| MU7147 | 196.1 | 200.3 | 185.2 | 173.5 |

Supplementary data – Table S3 : Size distribution (nm) of fraction 7-14 in LF Bulls

| LF Bull No | F7-8 | F9-10 | F11-12 | F13-14 |
| --- | --- | --- | --- | --- |
| MU7649 | 258.7 | 240.5 | 226.7 | 206 |
| MU7584 | 197 | 187 | 170 | 167 |
| MU7094 | 209 | 193.4 | 184.3 | 160.9 |

Supplementary data – Table S4 : Concentration of seminal EVs in different fractions of HF bulls

|  | F 7-9 | F 10-12 | F 13-15 |
| --- | --- | --- | --- |
| HF r1 | 6.43 × 10^11^ | 4.13 × 10^11^ | 2.84 × 10^11^ |
| HF r2 | 6.54 × 10^11^ | 6.82 × 10^11^ | 4.48 × 10^11^ |
| HF r3 | 6.21 × 10^11^ | 3.95 × 10^11^ | 4.99 × 10^11^ |

Supplementary data – Table S5 : Concentration of seminal EVs in different fractions of LF bulls

|  | F 7-9 | F 10-12 | F 13-15 |
| --- | --- | --- | --- |
| LF r1 | 4.38 × 10^11^ | 7.30 × 10^11^ | 5.92 × 10^11^ |
| LF r2 | 5.61 × 10^11^ | 7.74 × 10^11^ | 6.51 × 10^11^ |
| LF r3 | 3.57 × 10^11^ | 6.79 × 10^11^ | 6.27 × 10^11^ |

Supplementary data – Figure S1: SDS-PAGE gel image of HF and LF bull seminal EVs protein (Fraction 7-14)


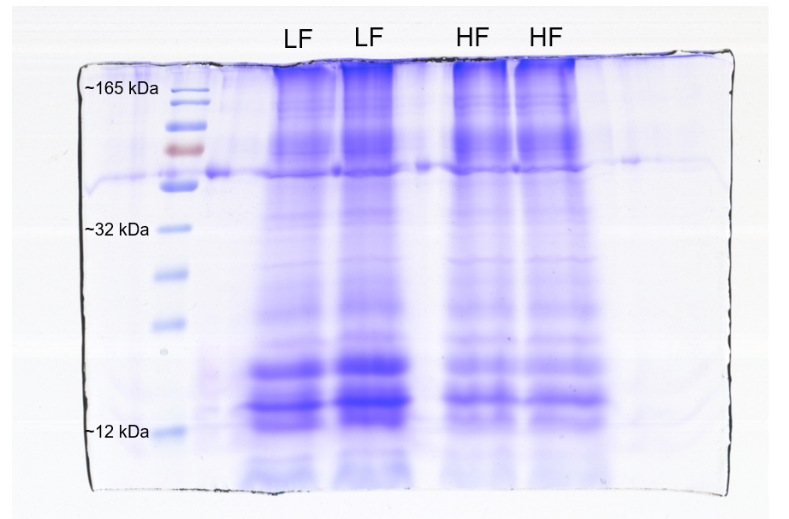


Supplementary data – Figure S2: Western blot results of CD63 and TSG101


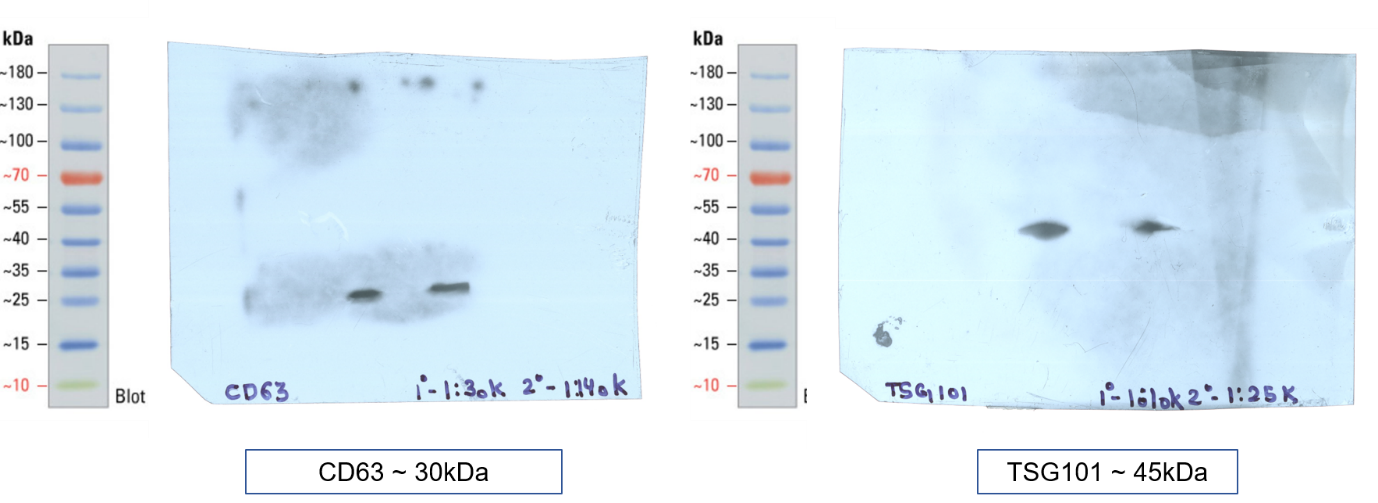


Supplementary data – Figure S3 : Secondary antibody controls for PDIA4 and GSN


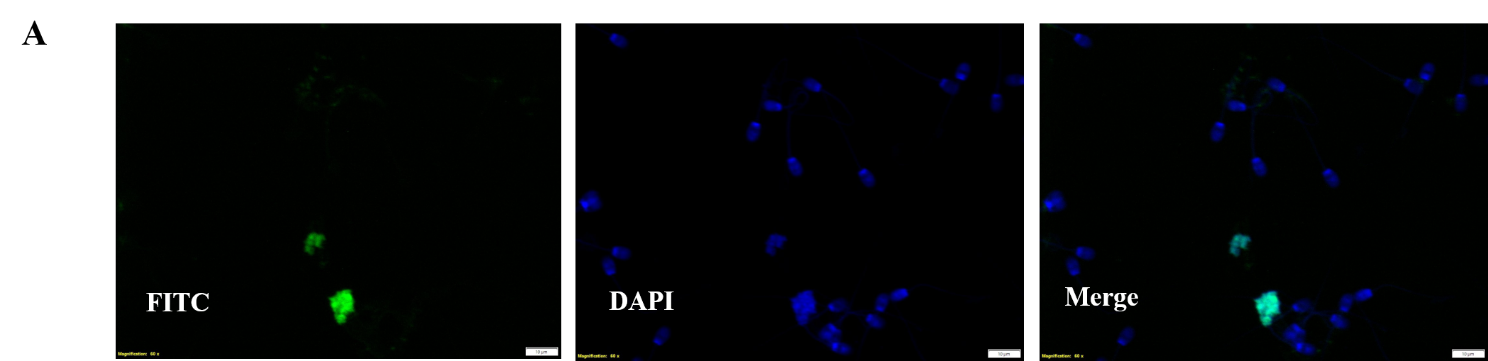


Supplementary data – Table S6 : Gene Ontology and pathway analysis of high and low abundant proteins in HF

1. Cellular component of high abundant proteins in HF EVs

| Term | Count | PValue | Genes |
| --- | --- | --- | --- |
| GO:0000786~nucleosome | 31 | 1.28E-40 | E1B9M9, F1MLQ1, E1BL10, F2Z4G5, E1B7N2, E1BL49, G3X807, F1MJU1, G3MXT2, F2Z4I6, F2Z4J1, A7MAZ5, P0C0S9, E1BLC2, A1A4R1, Q3ZBX9, P62803, E1BBP7, G3N2B8, G3N081 |
| GO:0005634~nucleus | 36 | 3.54E-06 | F1MLQ1, Q2NKV1, Q56JV9, Q3SWW9, E1B8Q8, A6QL75, F2Z4G5, F1MJU1, F2Z4I6, G5E526, F1N1I6, Q3T0R1, A7MAZ5, P45478, Q3SZ15, Q5I597, Q1LZE9, A5PKI0, Q5E9A6, F2Z4J1, P10152, P0C0S9, E1BLC2, F1MMR8, A1A4R1, Q3ZBX9, P62803, E1BBP7 |
| GO:0030125~clathrin vesicle coat | 3 | 5.87E-04 | P04973, F1N4F8, F1N579 |
| GO:0002102~podosome | 3 | 0.003972 | F1N1I6, A4FUG5, Q2KJA1 |
| GO:0001669~acrosomal vesicle | 3 | 0.031759 | G3N136, F1MR35, Q3SZW1 |

**2.** Biological processes of high abundant protein in HF EVs

| Term | Count | PValue | Genes |
| --- | --- | --- | --- |
| GO:0031507~heterochromatin assembly | 14 | 3.55E-20 | F2Z4J1, F1MLQ1, P0C0S9, F2Z4G5, A1A4R1, Q3ZBX9, F2Z4I6 |
| GO:0006334~nucleosome assembly | 17 | 1.28E-17 | E1B9M9, E1BL10, E1B7N2, E1BL49, G3X807, F1MJU1, G3MXT2, A7MAZ5, E1BLC2, P62803, E1BBP7, G3N2B8, G3N081 |
| GO:0006352~DNA-templated transcription, initiation | 9 | 9.38E-13 | E1B7N2, P62803, G3N2B8, G3X807, G3N081 |
| GO:0016191~synaptic vesicle uncoating | 2 | 0.028036 | Q08DX1, Q2KJA1 |

3. Molecular functions of high abundant protein in HF EVs

| Term | Count | PValue | Genes |
| --- | --- | --- | --- |
| GO:0046982~protein heterodimerization activity | 30 | 6.00E-29 | E1B9M9, F1MLQ1, E1BL10, F2Z4G5, E1B7N2, E1BL49, G3X807, F1MJU1, G3MXT2, F2Z4I6, F2Z4J1, P0C0S9, E1BLC2, A1A4R1, Q3ZBX9, P62803, E1BBP7, G3N2B8, G3N081 |
| GO:0003677~DNA binding | 31 | 4.10E-19 | E1B9M9, F1MLQ1, Q2NKV1, E1BL10, F2Z4G5, E1B7N2, E1BL49, G3X807, F1MJU1, G3MXT2, F2Z4I6, F2Z4J1, P10152, P0C0S9, E1BLC2, A1A4R1, Q3ZBX9, P62803, E1BBP7, G3N2B8, G3N081 |
| GO:0030527~structural constituent of chromatin | 3 | 0.002693 | A7MAZ5, A1A4R1, Q3ZBX9 |
| GO:0019899~enzyme binding | 5 | 0.007109 | P0C0S9 |
| GO:0005543~phospholipid binding | 4 | 0.007675 | A4FUG5, F1N4F8, F1N579, E1BK71 |
| GO:0008289~lipid binding | 4 | 0.0177 | Q08DX1, F1N4F8, Q2KJA1, F1N579 |

4. KEGG Pathways of high abundant protein in HF EVs

| Term | Count | PValue | Genes |
| --- | --- | --- | --- |
| bta05322:Systemic lupus erythematosus | 27 | 5.21E-29 | E1B9M9, F1MLQ1, P01030, F2Z4G5, E1B7N2, E1BL49, G3X807, G3MXT2, F2Z4I6, F2Z4J1, P0C0S9, E1BH06, A1A4R1, Q3ZBX9, P62803, E1BBP7, G3N2B8 |
| bta05034:Alcoholism | 27 | 1.13E-26 | E1B9M9, F1MLQ1, Q3SWW9, F2Z4G5, E1B7N2, E1BL49, G3X807, G3MXT2, F2Z4I6, F2Z4J1, P0C0S9, A1A4R1, Q3ZBX9, P62803, E1BBP7, G3N2B8 |
| bta04613:Neutrophil extracellular trap formation | 26 | 8.93E-25 | E1B9M9, F1MLQ1, F2Z4G5, E1B7N2, E1BL49, G3X807, G3MXT2, F2Z4I6, F2Z4J1, P0C0S9, A1A4R1, Q3ZBX9, P62803, E1BBP7, G3N2B8 |
| bta04217:Necroptosis | 14 | 7.26E-11 | F2Z4J1, F1MLQ1, P0C0S9, F2Z4G5, A1A4R1, Q3ZBX9, F2Z4I6 |
| bta05203:Viral carcinogenesis | 12 | 3.03E-07 | E1B9M9, F1N1I6, E1B7N2, P62803, E1BL49, G3N2B8, G3X807, G3MXT2 |
| bta04144:Endocytosis | 6 | 0.02167 | P04973, Q08DX1, F1N4F8, Q2KJA1, F1N579, Q5E9A6 |

5. Cellular component of low abundant proteins in HF EVs

| Term | Count | PValue | Genes |
| --- | --- | --- | --- |
| GO:0005829~cytosol | 315 | 1.25E-36 | P80227, Q3MHK9, F1MXB4, P31976, Q2HJ94, Q3ZCK9, Q1LZ81, P52556, Q2TBW7, Q3T077, A7MB50, Q95M59, Q0VCJ5, F2Z4I1, G3MXC8, A7MB57, F1MX22, F1MHC2, Q3ZCJ2, A5D7A2, Q3T178, F1MH34, F1MYV9, G5E6P3, Q2TA37, Q17QW3, Q0VCK0, E1BPK6, F1MUN7, Q3ZBX5, G3N1U2, F1MPD4, E1BME6, A7E3Q8, F1MIF2, A5PJB5, P00570, Q17QG2, E1BCS3, E1BKS0, Q5PXY7, P05307, Q08DR7, Q0V8F2, Q3T0I2, A7MBH9, Q08DB4, E1BJQ7, A4FV22, G3MZZ6, P15103, Q0III6, Q3MHN0, A6QLL8, E1BKB2, G5E628, G5E507, A6QNP1, P55052, F1N468, F1MMK2, P62935, P00442, Q05B62, F1MIC3, F1MZU2, Q2TBG8, F1MHR4, A7MBI5, G3N3K4, O97764, Q2KJ32, A7MBI0, Q58DK4, F1MJ59, Q58DK5, A7YY28, Q3T0H0, F1N1A3, Q2TBX2, Q0VCM0, F1MKX4, P68250, Q3MHL4, Q58CY6, E1BMG1, Q3T0Y8, P62261, Q0VCM4, P00435, P79251, F1MBF6, Q08DQ4, F1MZV1, A7E3S8, Q08E20, P27214, Q17Q89, A7MBJ5, Q3ZBM5, Q32L63, G5E5T1, P10279, Q9XSG3, O62829, F1MVS9, Q2KJD2, G3X757, F1MWU9, P61585, A7E3T7, P48644, F1N6Q0, E1BG49, P61223, F1N049, Q05927, F1MNT3, P31404, P31407, P11019, P31408, F1MM14, Q9N2J2, Q2KJE5, F1MBL2, Q5EAC6, F1MND1, G5E5R6, P21856, F1MND2, A0JNB0, Q1RMR9, O62830, P25417, G3MXH2, A0JN54, F1MBI1, F1MCK2, Q2KI42, Q17QK8, F1MNQ4, P17697, P16116, A8E4M7, A2VE46, F1MPE1, Q3SZC4, Q29RK4, F1MRY9, F1MN04, P49951, F1MRZ3, E1BP90, E1BP91, E1BFV0, O02675, Q5E9A3, A7E3X2, Q9XSJ0, A5PJM4, Q3SZB7, P41541, A1A4P5, Q0VCQ6, E1BPI3, F1N036, P04272, A5D7K0, F1MYX5, Q2NL31, P02694, F1MIM3, Q3SZU4, Q3SX02, A4IFU2, F1N6D3, Q5E9F9, Q58DR7, Q3T105, F1MXJ5, F1MF68, Q9TU25, Q28205, Q05204, Q5E964, Q5EA61, P62998, F1N647, Q2KIV7, F1N5S6, H7BWV5, F1MBP8, Q58DA0, E1BKZ9, F1N650, Q58D08, O77834, Q2KJ93, F1MW91, F1MSA1, E1BAV2, Q3MHR7, F1MD34, Q5BIP4, E1B7R3, Q3T0N1, A6QLB8, P10096, F1MLD7, Q0VD48, P67774, A4IFD0, Q1JPD9, E1BPC3, A5D7I1, E1BHT5, P81947, P52175, Q2KIY3, E1BBY7, F6R6Q1, A6H742, P35478, E1BNQ3, Q1RMX7, A3KMV2, Q9TU47, Q58DT6, Q5EAD0, P17248, Q863B3, Q2KJJ9, A4FUZ3, Q5E947, Q5E946, Q17R14, Q2HJ26, Q2HJ23, Q58DC0, P34933, G3MZS9, P81287, Q3SZ52, Q3T0P6, P13214, Q9BGI3, Q58CQ2, Q29455, Q2TBP0, Q9BGI1, Q3T000, F1MWH2, A5PJY9, F1N2L9, Q2HJ33, F6QVC9, Q5E956, F6Q087, F1MJS4, P46193, E1B7M8, A6H768, A3KN51, G3X7N4, Q3T145, P63243, Q95M18, Q32PF2, Q3T030, F1N443, P0CB32, F1N0M0, A6H772, Q3ZBU7, Q76LV2, Q3ZCF0, F2Z4F5, Q56JW4, Q3SX32, Q3ZCF3, A7MBC5, Q3ZBD7, Q27970, Q58CS7, Q27971, P56966, A5D984, Q27965, F1MWN1, Q148J6, P60661, A6QR28, Q3SZH7, E1BBT9, Q2HJ57, F1MJQ1, A5D7D1, P04896, Q0VCX2, Q3ZBG1, Q3T0E0, Q3ZCI4, Q28035, A7YW98, F1MBQ1, F1MFY7, Q0VCX5, Q27975, Q5E987, Q3ZC84, A4FV72, P61763, Q9XSA7, Q5EA87, Q5EA88, P19120, A4FV55, P84080, F1MLV3, Q3T035, P13135, A6QR46, P10790, P33097, F1MDW1, A6H7J6, Q2HJH1, P21671, F1N556, F1N5V9, F1MU79 |
| GO:0005737~cytoplasm | 377 | 1.17E-34 | Q3MHK9, O97859, P0CH28, F1MCZ3, Q4U5R3, Q2HJ86, E1BDS9, E1BAN2, A5D7A2, Q8WML4, E1BPK6, A5PKD6, A5PJB5, A6QPS1, Q58DH9, P50397, Q0VBZ0, Q0V8F2, Q3MHM5, G8JKX4, G3MZZ6, P15103, E1BNH2, Q0III6, P63103, F1MIC9, F1MBV2, E1BKB2, E1BPL8, G5E507, F1MIC3, Q2TBG8, A7MBI5, F1MTZ1, A7MBI0, A7YY28, F6Q9S4, P55859, Q3SWX7, A0JNI4, Q32LE9, E1BMG1, Q32LE5, G5E619, P62261, Q08DQ4, Q08E20, P27214, Q17Q89, A7MBJ5, A4FV08, G5E5T1, F1MYH5, G5E5T2, Q3SZA6, E1B8X4, Q0IIG5, G3X757, P05131, F1N049, Q2NL00, F1MNT3, P31407, Q3B7M9, P0CG53, F1MM14, F1MWE0, F1MZK4, Q3MHN5, Q5EAC6, G5E5R6, P21856, Q5EAC7, G3X6U1, Q3B7N3, Q2KJ44, Q2KIS7, Q9MZG3, Q58CN9, G3MWX2, P17697, Q3MHR0, A2VE46, P17453, P00921, Q1JPA0, F1MRY9, F6RP72, F1MBZ1, Q08DM8, E1BFV0, F1N0T3, O02675, Q3SZB7, A1A4P5, E1BPI7, P04272, Q1JQD4, F1MIH4, Q2KI48, P02694, A6QPZ0, P38409, Q3SX02, Q5E9F7, F1MN60, Q2KJH4, A1A4K3, P62992, P62871, F1ML28, P80177, E1BHC8, A4IFC6, Q148F1, A2VDS0, P10096, F1MZP0, P11064, A4IFD0, Q865V6, E1BA62, Q5E9D5, Q3SYU2, Q2KJJ9, Q95140, Q148G7, Q17R14, Q58DC0, P02584, O46414, O46415, Q3SZ54, P81287, G5E589, P13214, F1N6C4, P13696, Q2LGB5, P79098, Q2HJ33, Q17QQ3, F1MMT9, Q17QQ2, P46193, F1MJ28, F1MKU3, E1BLF1, P68138, F1N6I4, A5PK96, P62833, Q3ZBU7, Q5E9K0, E1BA06, P06623, A7MB28, F2Z4F5, Q56JW4, F2Z4F0, A5D984, Q08DW2, F1MWN1, Q148J6, A5D980, Q2HJ58, Q09430, Q07130, Q5EA79, Q0VCX2, G3X8B1, Q10741, E1BCW3, E1BD83, Q2T9S4, F2Z4C1, E1BP01, Q0VCX5, A5D973, A5D7T5, E1BA27, P26452, E1BCX9, Q2HJ81, Q58DG1, Q28046, P13135, Q32PI5, A5PK65, F1MGQ5, G3X7B2, E1BL29, F1MI43, A6QLZ0, Q17QE5, F1MW03, Q0VCJ5, G3N132, Q3T0X5, G3MXC8, F1MWQ2, G3X8C8, Q3ZCJ7, Q3T178, Q3T064, Q2TA37, E1BFH4, F1MUN7, A7E3Q8, P00570, Q17QG2, E1BKS0, Q5PXY7, P79136, F1MXQ5, Q0P5B0, G3N0T0, Q3T0Z7, P55052, P62935, P00442, F1MZ40, F1MZU2, P35507, F1MJV6, F1MJ59, Q2TBX2, Q0VCM0, Q2TBX4, P68252, P68250, Q2TBX6, F1MEL0, Q58CY6, Q3T084, F1MQD1, Q0VCM4, F1MX11, E1BMX0, G5E5C8, Q5E9B1, P83939, Q5E9B5, P10279, F6Q3P6, Q9XSG3, Q5E9B7, E1BNS9, Q2KJD0, F6RJ91, F1MWU9, Q29RI6, A7MB90, P02769, Q3ZBM0, E1BFC8, P11017, Q2KJE7, F1MBL2, F1MND1, Q1RMR9, P25417, Q3SZP7, P23356, F1MBI1, F1MCK2, E1B7G3, Q17QK8, Q3ZC07, Q17QK3, P79334, F1MI60, E1BM27, Q8SQ21, Q0P5F3, F2Z4K0, F1MPE5, Q148C9, E1BP91, Q32L99, P20072, Q5E9A3, Q9TR36, A7E3X2, Q9XSJ0, A5PJM4, E1BHJ0, Q0VD27, A5D7K0, Q3SZ18, F1MYX5, Q3SZ19, Q2NL31, G3MZP2, Q2KIW6, Q0IIC1, Q9XSC6, P63048, P62194, E1BG08, Q2NL29, F1N647, Q2KIV7, F1MBP8, F1MVX2, F1N650, O77834, Q2KJ93, F1MW91, A6QLB7, E1B953, Q58DQ3, A6QLB8, F1MQN0, P68509, P81948, P81947, Q2KIW9, Q0P594, P52175, A6H742, A3KMV3, Q9TU47, Q58DT6, A3KMV5, Q5EAD0, P17248, Q863B3, A4FUZ3, Q5E946, F1MX83, F1MU24, F1ME38, P34933, G3MZS9, Q5KR48, A6QLD6, Q5EAE3, Q58CQ2, Q2TBP0, Q9BGI1, F1MNF8, G5E6J5, F6QVC9, F1MTR1, F1MU34, G3MY14, P33672, F1MC48, Q3T024, P63243, Q3T030, E1BIQ8, F1MEZ5, P0CB32, Q3SZI4, Q1JPG7, F1N2Q7, Q32KL2, O18824, Q76LV2, Q32LP2, Q3ZCF0, Q3ZCF3, Q3ZBD2, Q58DU5, Q27970, Q27971, P56966, Q27965, Q27966, A6QR28, Q3SZH7, A5D7D1, F1MB08, B0JYP8, Q3ZBG0, F1MT40, Q08DF4, Q27975, Q2YDJ4, Q3T052, A4FV72, Q5E984, Q3SZK8, Q32KN8, Q9XSA7, P20456, P80724, P19120, Q3T035, P10790, F1MDW1, P00517, Q58DW2, P00514, E1BH17, P00515, F1MU79 |
| GO:0016324~apical plasma membrane | 59 | 1.73E-22 | P46193, A5PJL8, F1MXB4, G3MWR4, P31976, Q58DR7, F1N6D4, F1MD24, Q17QE5, Q0IIG8, Q27960, Q3T0X8, Q2HJ49, Q0IIG5, E1BKL9, A7MBA9, Q08DL0, F1MXW4, Q3T0C6, Q4GZT4, E1BM92, F1MDL3, P81425, Q32LP2, B8Y9S9, Q3ZCJ2, P31404, P31407, Q9XT96, F1N650, P11019, P31408, Q9XT97, E1BQ12, F1MK52, F1MRH1, G5E5R6, E1B9W1, A3KMY4, Q8WML4, P58353, Q3MHW6, E1BPC3, O18875, P07688, G5E5A9, A7MBD8, Q2T9S6, F1N2B5, E1BGK6, Q3ZC83, Q3SZK8, F1MEP6, F1N5Z0, F1MLR4, Q17QL5, Q3ZCH8, P13214, F1MI32, E1BQ28, A2VE13, Q95135, E1BIH1, F1N6H1, P79251, F1MIH4 |
| GO:0042470~melanosome | 34 | 2.35E-22 | Q865V6, Q0VCX2, P05307, Q0VCV6, Q9XSK2, Q863B3, Q95M18, P63103, Q05204, G5E507, Q95121, P62998, P49951, F1N647, Q3T0C6, Q2T9X2, P06623, F1MDL3, P81623, Q76LV2, Q3ZBD1, P19120, P31408, Q58DS9, P68250, Q3MHL4, P38657, A5D7E8, F1MI32, Q29455, F1N3H1, P62261, A6H7J6, F1MUN7, P04272, P07688, P27214 |
| GO:0005794~Golgi apparatus | 93 | 5.80E-14 | F1MI46, P07107, Q2KIW7, Q2KJ81, A5D7R9, A7MB57, F1N647, P34955, E1BKZ9, F1MW91, F1MSA1, O02853, Q3T0N1, F1N3H1, Q2TA37, E1BIN5, E1BPK6, G3N1U2, Q08DS7, F1MIF2, F1MPT8, F1N6V7, A6QLS9, Q2KIY3, Q32L40, A4FV22, A3KMV2, Q3T133, Q03247, Q05B62, E1BHU3, F1MTZ1, Q3T0P7, P12763, Q58DS5, Q29455, F1MBF6, Q08DQ4, F1MT12, Q3SZF2, P08037, A7MBJ5, F6Q3P6, P10279, Q58D55, Q0IIG8, Q95122, Q32LF7, A7MBA9, Q58DV1, P02769, F1N443, F1MM97, Q2KHZ8, A5PKI3, Q3ZBD1, G3X7G4, Q9XT97, Q148J4, Q27971, P21856, G3MXH2, E1BC58, G3MYT7, F1MJQ1, Q10741, F1ML49, Q58CN9, Q17QK3, F1MXL8, A7MAZ2, Q5EA87, Q5E9I4, E1BPZ1, Q5EA01, P81127, F1N672, Q5E9I6, Q17QL5, Q9TR36, A0JNM2, A4FV55, F1MKT0, Q70E76, P84081, P84080, F1MEX9, F1MVI9, A7YW22, A6QR46, Q0VCQ6, A7Z024, F1N6H1, Q2HJH2, F1N554, F1MHF1, F1MIH4, E1BLA8, F1N5V9 |
| GO:0070062~extracellular exosome | 24 | 1.14E-13 | P46193, F1MCK2, E1BBY7, G5E5A9, A4IFU2, A3KN51, Q5E9B7, Q58D31, Q03247, A6QLL8, P02769, F1MY28, F1MNI4, B8Y9S9, F1MM32, F1N650, A5D7A2, F1MK52, A4IFN6, Q08DW0, Q1JPD9, Q3SZJ7, P79098, G3MXJ5, A6QM01, P63258, F1MUL0, Q2TBH7 |
| GO:0005615~extracellular space | 140 | 1.84E-13 | F1MI46, A5PJL8, A6QPZ4, A2I7N0, A6QLZ0, A2I7N1, Q2T9M7, F1MAV0, Q32LB5, G5E604, Q9XSC6, Q2KIF2, P26779, Q5EA61, Q9XSC9, F1MMM6, P34955, G3MYW7, F1MSZ6, F1N650, P80177, P28291, F1MEV7, A3KMY8, O02853, F1MPL6, F1MYN5, G3N1U4, F1MNH9, F1MSR1, A6QNW8, P60986, Q58DL9, Q865V6, G5E5A9, F1MMS7, E1BF81, G5E5A7, Q3T0I2, Q3T004, Q0IIA2, Q03247, P37141, F1MET0, Q6QRN6, P55052, E1BG25, F1MX83, F1MKG2, A6QP36, F1MZ40, Q3ZBS7, A7MBI5, F1MNV5, F1MM32, O46375, F1N1Z8, G3X6K8, P50448, Q0VCM0, Q3T0P6, Q3T0P7, G3MX66, F1N3E9, P12763, Q0VCU3, Q2T9N7, F1MKH8, G8JKW7, P24627, F6RF06, Q08DQ4, G3MZ19, P08037, P46193, E1BI82, P04557, A3KLR9, G3N0F4, P31098, F1MLW2, Q58D55, G3X7N4, F1MVK1, F1MVS9, F1N1G1, Q95121, F1MB31, P02769, Q3ZBM0, F1MM97, P06623, Q2KHZ8, A5PKI3, B8Y9S9, Q7SIH1, O02659, Q3ZEJ6, Q3MHN2, F1MCV8, E1BFC8, G1K200, Q3ZBD7, E1BMJ0, F1MGN0, E1BNL3, Q3MHN5, A5D7E8, P00735, Q1JQB0, P00974, P28800, F1MUR6, P07688, Q2KIS7, P06833, Q17QK3, Q2TBU0, Q9N2I2, A7E3W2, P17697, Q0VD19, F1MPE1, P17453, Q5E984, Q0VCH9, F1MNQ8, P00760, A6QPP2, E1BPZ1, F1MRZ5, F1N0T3, F1MRZ6, Q3ZCH5, F1N3A1, E1BLR9, A7YW22, P38657, F1N3J3, G3X701, A5PK65, P04272, F1N0T7, E1B8B5, F1MX50, P01045 |
| GO:0005839~proteasome core complex | 15 | 3.03E-13 | F1MBI1, Q3ZBG0, E1BD83, Q3ZCK9, Q3T108, P33672, G5E589, Q2TBX6, Q58DU5, Q2TBP0, Q5E987, Q3T0Y5, Q3MHN0, Q3T0X5, Q2YDE4, Q5E9K0, Q32KL2 |
| GO:0005902~microvillus | 20 | 1.20E-11 | Q3MHK9, E1BM92, Q32LP2, P31976, P31404, P31407, P11019, P31408, Q58DR7, E1BHC8, A6QLD6, A7YW22, F1MK52, Q27966, Q2HJ49, E1BPK6, Q3SZK8, F1MTR1, Q17R14, Q9XSA7, Q3SZP7 |
| GO:0005764~lysosome | 40 | 1.61E-11 | F1MJQ1, Q17QK3, Q3MI05, Q3T0I2, Q0P5F0, Q58D55, Q2TBW7, Q08DD1, Q0VD19, F1N1G1, P26779, E1B9E8, G5E5M8, P49951, Q05B62, O18824, F1MBJ3, Q2KHZ8, F1MHR4, P31404, E1B9H0, E1BKZ9, F1MPT2, O77834, P56541, P19120, F1MSA1, A4IFC6, E1B7R3, Q27970, Q0VCU3, A3KMY8, Q27971, Q3T0Y8, F1MAU4, F1MT12, A6QM01, H7BWW2, Q58DH9, E1BHP0, P07688, Q2KIM0 |

6. biological processes of low abundant proteins in HF EVs

| Term | Count | PValue | Genes |
| --- | --- | --- | --- |
| GO:0006096~glycolytic process | 18 | 1.38E-12 | F1MB08, E1BCS3, F1MIM3, E1B959, Q3T0P6, Q32KV0, Q3SZ62, Q3ZBD7, Q2KJE5, G3X7N4, Q3T014, A5D984, P10096, F1MX69, A6QLL8, E1BME6, F1MZV1, Q5E956, Q1JPG7 |
| GO:0006457~protein folding | 30 | 3.21E-11 | Q17QG2, Q2HJ94, G3X8B1, P05307, Q32L40, Q3ZCI9, Q3T0K2, F1MWD3, A7MB50, Q95M18, Q0III6, Q28205, F1MGX0, G5E507, A4FV72, F1N6Q0, P62935, Q2T9X2, Q76LV2, Q3MHL7, Q3ZBH0, F1MM32, G3MZS9, Q2TBX2, Q3T0L2, P38657, A5D7E8, Q5EAC6, Q2TA37, A1A4P5, Q3T084, A6H7J6, A7Z066, G3X861, Q17Q89 |
| GO:0006508~proteolysis | 36 | 1.90E-10 | P80227, P83939, G3N0F4, Q17QK3, Q3MI05, Q3T0I2, Q2TBU0, Q08DB4, F1MXQ5, Q3T0X8, Q2T9M7, E1BPL8, Q3ZC84, F1MB31, Q5E946, F1N0M0, F1MEA3, P00760, P81425, E1BP91, O02659, P81187, G3X6K8, F1MCV8, Q27970, Q27971, F1MKH8, P24627, F1MQD1, Q2HJH1, Q3SZH7, F1MAU4, P00735, P79098, F1MSR1, P60986, F1MU34, Q3SZ19 |
| GO:0010498~proteasomal protein catabolic process | 16 | 2.87E-10 | F1MBI1, Q3ZBG0, E1BD83, Q3ZCK9, Q3T108, P33672, G5E589, Q2TBX6, Q58DU5, Q2TBP0, G3X757, Q5E987, Q3T0Y5, Q3MHN0, Q3T0X5, Q2YDE4, Q5E9K0, Q32KL2 |
| GO:0015031~protein transport | 46 | 1.47E-09 | A5D7D1, F1MXB4, P50397, Q32L63, Q08DR7, F1MXJ5, A3KN51, Q9XSK2, Q0IIG8, Q0P5B0, A6QR35, Q2KJD2, Q0P5F3, Q0VC16, A7MBA9, E1BHS9, F1MN49, Q3ZBW5, P61763, Q17R14, F1MHC2, F1MZU2, E1BP90, Q3T0D2, Q2KJ32, Q9XT97, F1MSA1, Q58DS9, Q58CS7, Q3T178, Q58DS5, Q6EWQ7, Q3T0N1, Q3T000, Q27966, P21856, Q58DW6, A6QR46, A4IFN6, Q3T0Y8, Q0VD48, E1BPK6, F1N6H1, E1BPI7, Q32LM6, Q58DF6, Q2TBH7 |
| GO:0006886~intracellular protein transport | 44 | 6.19E-09 | F1MXB4, E1BMW2, Q3ZBM5, Q17R06, Q3ZBG1, Q2KJ81, F1MT40, Q1LZ81, Q2TBW7, F1MF68, G3N3N1, Q0IIG8, Q3T133, A7MAZ2, P61763, F1MNQ8, Q3ZBS3, P49951, F1MHC2, F1MNI4, Q05B62, F1MZU2, F1MDL3, P81127, P81126, Q3ZBD1, Q5E9I6, G3X7G4, A8E4N0, P84081, P84080, Q5BIP4, A5D7S0, P41541, F1MWN1, A6QR46, Q0VCK5, Q2HJH2, Q08DS7, F1MBF6, P68509, Q3ZBT5, F1MIF2, Q5E9R3, Q3SZF2 |
| GO:0007339~binding of sperm to zona pellucida | 16 | 7.35E-09 | Q2T9X2, Q3ZBH0, E1BLW6, Q32L40, A6QPE3, Q3T0K2, F1MWD3, F1MI43, Q08DD1, Q32LB5, A6QLL8, P0CB32, F1N369, F1MFC4, E1BG77, P08037 |
| GO:0005975~carbohydrate metabolic process | 27 | 1.97E-08 | F1MJS4, E1BBU8, G5E5C8, Q2KIS1, F1N6Y1, A4FV08, Q5E9B1, E1BGB7, F1N2B5, Q58D55, Q3T145, E1BNS9, Q32LF7, Q2KJJ9, Q08DL0, E1B9E8, Q5EA87, E1BDR2, Q5EA88, F1MVX2, E1B9H0, F1MTV1, G3MY87, F1MZM9, A3KMY8, Q3SZB7, F1MZP0, F1MND2, H7BWW2 |
| GO:0016192~vesicle-mediated transport | 33 | 2.80E-08 | F1MXB4, E1BMW2, P50397, Q2KJ81, F1MXL8, F1MF68, G3N3N1, Q2KJD2, Q27975, F1MWU9, P0CB32, P61763, F1MNQ8, Q3ZBS3, P49951, F1MHC2, P34933, P81127, P81126, Q5E9I6, G3X7G4, P19120, A8E4N0, P84081, P84080, Q27965, A5D7S0, P21856, A4IFJ2, A6QR46, Q0VCK5, Q08DS7, Q3ZBT5, F1MIF2, Q3SZF2 |
| GO:0006897~endocytosis | 28 | 5.35E-08 | E1BN81, G3X7B2, P52175, E1BA62, A7E3W2, Q08DF4, Q2TBW7, Q0VC16, A7MB90, F1MNI4, F1MM97, E1BPZ1, E1BKZ9, Q3ZBD1, Q2KJ93, G3X7G4, F1MW91, O02675, A7E3X2, G1K200, F1MND1, A4IFN6, A1A4J6, E1BPK6, G3N1U2, Q08DS7, G3MXH2, Q5E9R3, A5D7I1, G3N0B5, E1BJV0 |

7. Molecular functions of low abundant proteins in HF EVs

| Term | Count | PValue | Genes |
| --- | --- | --- | --- |
| GO:0003924~GTPase activity | 77 | 2.38E-26 | G3MZP2, Q3ZCK2, P38409, Q5EA19, P38408, E1BED8, Q0IIG8, Q9TU25, Q2KJD0, P62871, F2Z4I1, P61585, Q1RMI2, P62998, P61223, Q2HJ86, A5D7R9, Q3ZBU7, Q3ZBD1, Q2KJ93, F1MW91, Q3ZCJ7, P11017, Q148J4, E1B953, Q3ZCA7, F1MND1, G5E6P3, G8JKY9, Q2TA37, Q1RMJ6, G3N1U2, G3MXH2, P81948, G3N0B5, P81947, A6QLS9, E1BC58, F1MJQ1, P04896, Q17R06, F1N461, Q3ZBG1, E1BA62, F2Z4C1, A7MBH9, E1BNQ3, Q3SYU2, Q3MHM5, G5E631, A5PKL2, Q08DF4, Q32PH8, G3N3N1, F1MNI8, F2Z4K0, Q3ZBW5, Q32KN8, F6RP72, A5D977, E1BA29, F1MNI4, Q5E9I6, Q2HJ81, Q5E9M9, Q58DS9, P84081, P84080, Q58DS5, A6QR46, Q58DW6, G5E619, Q2HJH2, F1MNF8, G5E6J5, F1MBF6, Q3SZF2, Q2TBH7, E1BMX0, F6Q087 |
| GO:0005525~GTP binding | 80 | 3.41E-23 | G3MZP2, Q3ZCK2, P38409, A4IFU2, Q5EA19, P38408, E1BED8, Q0IIG8, Q9TU25, Q2KJD0, F2Z4I1, P61585, Q1RMI2, Q58DV1, P62833, P62998, P61223, Q2HJ86, A5D7R9, Q3ZBU7, Q3ZBD1, Q2KJ93, F1MW91, Q3ZCJ7, Q148J4, E1B953, Q3ZCA7, F1MND1, G8JKY9, Q2TA37, Q29RJ1, Q1RMJ6, G3N1U2, G3MXH2, P81948, G3N0B5, P81947, A6QLS9, E1BC58, F1MJQ1, P04896, P52175, Q17R06, F1N461, Q3ZBG1, E1BA62, Q0V8F2, F2Z4C1, A7MBH9, E1BNQ3, Q3SYU2, Q3MHM5, A5PKL2, Q08DF4, Q32PH8, G3N3N1, F1MNI8, F2Z4K0, Q3ZBW5, Q32KN8, F6RP72, A5D977, F1MNI4, Q5E9I6, Q2HJ81, Q5E9M9, Q58DS9, P84081, P84080, Q58DS5, A6QR46, Q58DW6, G5E619, A7Z024, Q2HJH2, F1MNF8, Q2HJ33, G5E6J5, F1MBF6, Q5E9R3, Q3SZF2, Q2TBH7, E1BJV0, E1BMX0, F6Q087 |
| GO:0005524~ATP binding | 165 | 3.79E-19 | Q2KIW6, Q2HJ94, G3X7B2, F1MIM3, Q5E9F9, F1N7F3, G3MW11, D3IVZ2, F1MN60, P00829, Q9XSC6, G3N132, P62194, F1N3G6, Q5EA61, A7MB57, F1MHK9, F1N5S6, E1BAN2, Q4GZT4, E1BCR1, Q3ZBH0, G3X8E2, Q2HJB1, A3KN22, A5D7A2, A6QLB8, F1MYV9, G3MZH7, A1A4J6, F1N0B2, Q0VD48, E1BPK6, A4IFD0, E1BME6, E1BPC3, Q2KIW9, F1N0J3, F1MS16, P00570, F1MBM5, E1BN81, E1BCS3, Q0VBZ0, P52175, E1BBY7, F6R6Q1, Q3SZ65, Q32L40, Q0V8F2, E1BNQ4, G8JKX4, G3MXV4, G5E631, P15103, F1N7D5, P17248, A3KMV5, A4FUZ3, F1MGX0, F1MNE5, F1MBV2, G5E507, G3X696, Q17R14, A6QLT9, Q2HJ26, F1MIC3, F1MZU2, P35507, F1MHR4, F1ME38, Q17QH2, P34933, Q3MHL7, Q58DK4, E1B8N5, Q3SZ54, Q3T0P6, F1MUC1, Q2TBX4, Q3T0P7, A6QLD6, A0JNI4, Q0P5K3, F1MI32, P13696, E1BIH1, Q3T084, Q0VCM4, G3X861, Q2HJ33, F1MZV1, G3MY14, A6H768, Q5E9B5, F6Q3P6, E1B748, Q3T0K2, E1B7U7, F1MWD3, G5E5T3, G3X7N4, G5E5T2, Q95M18, F6RJ91, Q0IIG5, G3X757, P68138, Q32PF2, F1MWU9, F1MC51, A7MB90, A7E3T7, Q3T030, Q32S38, F1N6Q0, P0CB32, F1MQ01, E1BG49, F1MXW4, P05131, Q1JPG7, F1N049, F1MFC4, Q2T9X2, Q76LV2, P31404, P31407, P31408, E1BM99, F1MWE0, A7MBC5, E1BQ12, Q3ZCF7, A5D984, Q27965, Q5EAC6, Q27966, A0JNB0, Q2KJ44, P63258, Q2HJ58, E1BBT9, A0JN54, Q0VCX2, Q3ZC07, E1BCW3, F1MI60, A7YW98, Q3ZCI9, E1BP01, A8E4M7, Q27975, G3X810, A5PK75, E1BIS8, F1MDE3, F1MN04, A5PKG9, F1N4K1, F1N272, G3MW08, P19120, A7E3X2, E1BPI3, F1MDW1, P00517, F1N7J2, G3X6W2, Q5E9R3, E1BJV0 |
| GO:0005515~protein binding | 48 | 6.01E-16 | P10279, Q3MHX6, A1A4K3, A7MB50, F1MAV0, P00829, P62871, Q28205, P02769, P62998, A6H772, F1N049, Q76LV2, B8Y9S9, Q3ZBH0, P0CG53, O02853, P10096, Q2TA37, Q0VCK5, P00735, P00974, P81948, Q0P594, Q10741, G5E5A9, P79136, Q3T0I2, Q08DF4, P63026, P61763, P00442, P00760, P19120, F6Q9S4, O02675, P84080, P12763, Q3T035, F1MI32, Q29455, P24627, P00517, P00514, P04272, F1N036, Q17QQ3, Q5E9R3, P08037, F1MU79 |
| GO:0051082~unfolded protein binding | 30 | 1.59E-14 | Q17QG2, Q0VCX2, Q2HJ94, Q32L40, Q3ZCI9, Q3T0K2, F1MWD3, P17697, Q95M18, Q27975, Q0III6, F1MWU9, F1MGX0, G5E507, A5PK75, F1N6Q0, P0CB32, Q2T9X2, Q76LV2, P34933, Q3MHL7, Q3ZBH0, P19120, Q2TBX2, Q2TBX4, Q27965, Q5EAC6, A1A4P5, Q3T084, A7Z066, G3X861, Q17Q89 |
| GO:0016887~ATPase activity | 53 | 2.02E-13 | Q2KIW6, Q5E9F9, Q0VCV6, E1B748, Q3T0K2, F1MWD3, D3IVZ2, P00829, Q95M18, G3X757, F1MWU9, P62194, Q3T030, P0CB32, F1MQ01, E1BAN2, Q2T9X2, Q76LV2, E1BCR1, Q3ZBH0, G3X8E2, Q2HJB1, F1MWE0, Q27965, Q0VD48, A1A4J6, E1BBT9, Q09430, F1MBM5, Q0VCX2, E1BBY7, Q3SZ65, Q32L40, Q0V8F2, Q3ZCI9, G5E631, Q27975, A4FUZ3, F1MGX0, F1MNE5, G5E507, A5PK75, F1MZU2, P34933, F1N272, Q3MHL7, Q3SZ54, P19120, Q2TBX4, F1MI32, Q3T084, F1N7J2, P09487, G3X861, Q2HJ33, P79251, G3X6W2 |
| GO:0005200~structural constituent of cytoskeleton | 21 | 9.41E-13 | E1BLT3, Q2HJ81, Q3MHR7, Q3ZCJ7, F2Z4C1, Q3T035, Q3MHM5, F1MEL0, E1B953, Q2KJD0, F2Z4K0, G3MXC8, F1MNF8, G5E6J5, Q32KN8, P81948, F6RP72, F1N049, Q2HJ86, P81947, Q3ZBU7 |
| GO:0000287~magnesium ion binding | 42 | 1.20E-12 | F1MJS4, G3MY14, E1BN81, F1MB08, P52175, Q2T9S4, Q1LZ81, P19111, E1BNQ3, Q9XSG3, O62829, Q6B855, G5E5T2, G3N0T0, F6RJ91, F2Z4I1, A7Z014, F1MBV2, F1N3G6, Q58DV1, Q32S38, F1MXW4, P05131, Q1JPG7, F1MFC4, Q2KIV7, A6QQ11, P20456, E1BA06, A0JNI4, A2VDS0, F1MYV9, A5D984, Q08DW2, A1A4J6, F1N0B2, O62830, P00517, A7Z024, Q0VD27, E1BPC3, Q2HJ58, Q3SZ18, F1N0J3 |
| GO:0051015~actin filament binding | 40 | 2.14E-11 | Q3MHK9, A5D7D1, Q865V6, P31976, P79136, A6H742, Q5E9F7, Q5E9D5, E1BN47, F1N7F3, F1MC48, E1BM27, A6QR15, Q0III9, Q2KJH4, A4FUA8, F1MCZ3, G3MXC8, Q3B7M5, Q17R14, F1N049, Q5E9E2, Q28046, E1BHC8, Q3MHR7, Q5KR48, A6QLD6, Q148F1, Q58CQ2, F1MEL0, Q3T035, Q27966, Q148J6, E1BPK6, Q5E997, A7E3Q8, F1MTR1, Q3B7N2, Q3SZP7, F1MYX5, Q2HJ57 |
| GO:0019003~GDP binding | 22 | 4.37E-11 | F1MJQ1, Q3ZCK2, Q17R06, Q3ZBG1, A4IFU2, E1BNQ3, Q58DS9, Q148J4, A5PKL2, G3N3N1, Q0IIG8, Q3ZCA7, Q2TA37, F2Z4I1, G3N1U2, G3MXH2, A5D7R9, P61223, A5D977, Q2TBH7, F1MNI4, A6QLS9, E1BMX0 |

8. KEGG Pathways of low abundant proteins in HF EVs

| Term | Count | % | PValue | Genes |
| --- | --- | --- | --- | --- |
| bta03050:Proteasome | 33 | 2.813299 | 1.90E-25 | F1MBI1, Q2KI42, Q3ZBG0, Q2KIW6, E1BD83, Q5E9F9, Q3ZCK9, Q3T108, P33672, F1MYH8, A4FUZ3, Q5E987, P62194, Q3MHN0, Q3T030, Q3T0X5, Q5E964, Q4U5R3, Q5E9K0, Q32KL2, P56701, Q58DA0, Q3ZBD0, F1MWE0, A7MBA2, F1MKX4, G5E589, Q58DU5, Q2TBX6, Q2KJE7, F1MXE4, Q2TBP0, Q3T0Y5, Q2YDE4, Q2KJ46, Q3SZ19 |
| bta04144:Endocytosis | 68 | 5.797101 | 6.38E-21 | F1MGQ5, Q3ZBM5, Q32L63, F1N7F3, F1MXJ5, E1BKM4, A3KN51, Q2TBW7, Q0VCN9, F1MWU9, P61585, G3MXC8, P0CB32, F1N049, F1N371, G3X8E2, Q2KJ93, G3X7G4, F1MW91, Q2HJB1, Q3MHR7, Q3T178, Q58CS7, Q3T0N1, Q27965, F1MND1, G5E5R6, F1MPL6, Q0VCK5, Q148J6, Q0VD48, G3N1U2, Q08DS7, A5D7I1, G3MXH2, A6QLS9, F1MJQ1, E1BMW2, P79136, Q08DR7, E1BA62, F1N6J8, Q08DF4, G3N3N1, A8E4M7, E1BJL8, A4FUA8, Q27975, P49951, Q08E32, F1MNI4, Q05B62, E1BP90, P34933, Q5E9I6, P19120, Q148L0, Q58DS9, P84081, P84080, Q58CQ2, Q3T035, Q5E997, F6RF06, Q5E9R3, Q3SZF2, Q2TBH7, E1BJV0 |
| bta01100:Metabolic pathways | 205 | 17.47656 | 9.79E-16 | Q3ZCK3, O97859, P52556, Q08DD1, F1MCZ0, G3X8C8, Q3ZCJ2, F1MRH1, E1BFH4, G3MZH7, Q0VCK0, E1BME6, A6QPS1, Q58DH9, P00570, P15103, G3N0T0, E1BNH2, Q3T0Z7, A6QLL8, F1MBV2, P37141, A6QNP1, F1N468, Q3B7M2, F1MMK2, F1MHR4, Q2KJ32, F1N3V0, Q58DK4, A3KN04, F1MJ59, Q58DK5, P55859, A0JNI4, G3MY87, A7MBI8, Q3MHL4, Q58CY6, Q32LE5, F1MBW3, Q0VCM4, P00435, P09487, P79251, F1MZV1, Q08DQ2, Q08E20, P08037, G5E5C8, Q17QI3, Q2KIS1, A4FV08, Q5E9B1, G5E5C9, Q9XSG3, F1ME02, E1B7U7, E1BNS9, F1N1G1, F6RJ91, Q0IIG5, Q32LF7, F1MX69, A7E3T7, Q32S38, E1BNB2, P48644, F1MXW4, Q05927, Q2NL00, P31404, P31407, Q3B7M9, P11019, P31408, Q32KV0, Q9N2J2, Q2KJE5, F1MND2, Q29RJ1, F1MPH8, G3X6U1, A6QM01, A0JN54, Q17QK8, O46563, P79334, Q0P5F0, P16116, Q0VD19, A8E4M7, A7Z014, P00921, Q1JPA0, F1MRY9, F1MN04, F1MZY2, F1N4K1, E1BDR2, Q0P565, Q5EA01, A6QPY0, P21282, Q3SZB7, Q0VD27, F1MHF1, A5D7K0, Q2KJ66, F1MUP1, Q3SZ18, Q2NL31, Q2KIW7, F1MIM3, Q3SZU4, Q58DR7, Q3T105, P00829, Q0IIC1, Q9XSC6, P61420, Q5EA61, E1B9E8, Q2NL29, F1N647, F1MVX2, Q58D08, O77834, P80177, F1MSA1, A3KMY8, O02853, P10096, F1MZP0, P11064, P23956, A4IFD0, H7BWW2, Q2KIW9, F1N6V7, F6Q751, P52175, F6R6Q1, Q3SZ62, Q1RMX7, Q5EAD2, A4FUZ1, Q2KJJ8, Q2KJJ9, Q58D31, F1MET0, Q2HJ26, E1BHU3, F1MU24, E1B9H0, Q3T0P6, P39942, F1MZM9, F1MWH2, F1N2L9, P79098, F1N632, Q5E956, F1N6T5, G3MY14, F1MJS4, F1N6Y1, Q3MHG3, A6H768, Q3SX46, F1MJ28, Q0VCV6, Q6B855, Q28017, Q58D55, G3X7N4, Q3T145, Q32PF2, A5PK96, Q1JPG7, Q2KHZ8, Q56JW4, Q1JP75, Q3ZBD7, G8JKV7, E1BQ12, P56966, Q3T014, A5D984, A6QR28, Q3SZH7, F1MCF5, Q2HJ58, G3MYT7, E1BBU8, Q07130, F1MB08, Q5EA79, B0JYP8, E1BCW3, Q2T9S4, Q3ZCI4, Q28035, O77588, P19111, Q3ZC84, A6QQ11, Q5EA87, P20456, Q5E9I4, F1MTV1, Q70E76, Q148L6, F1MEX9, P33097, F1MDW1, Q58DW2, E1BH17, F1N554, Q1RML9, P21671, G3N2D8 |
| bta05020:Prion disease | 64 | 5.456095 | 2.11E-15 | Q2KIW6, Q5E9F9, Q3ZCK9, P10279, Q3T108, P33672, F1MYH8, Q9TU25, Q2KJD0, P00829, F1MWU9, P62194, Q3T0X5, Q3T030, Q5E964, P62998, P0CB32, P05131, Q2HJ86, Q3ZBU7, Q5E9K0, Q32KL2, Q58DA0, Q3ZBD0, Q3ZCJ7, Q3MHN2, F1MWE0, F1MD77, Q58DU5, E1B953, Q27965, A0JNB0, P81948, P81947, Q2KJ46, Q2KI42, F1MBI1, P32007, Q3ZBG0, Q0VCX2, E1BD83, F2Z4C1, Q3MHM5, Q27975, A4FUZ3, Q5E987, Q3MHN0, F2Z4K0, Q32KN8, P00442, A5D7T5, F1MIC3, P56701, P34933, Q2HJ81, P19120, A7MBA2, G5E589, Q2TBX6, F1MXE4, Q2TBP0, Q3T0Y5, P00517, F1MNF8, Q3ZBZ8, Q2YDE4, Q3SZ19 |
| bta05012:Parkinson disease | 63 | 5.370844 | 7.89E-15 | Q2KIW6, Q5E9F9, Q3ZCK9, Q3T108, P33672, P0CH28, F1MYH8, Q2KJD0, P00829, P62992, P63048, P62194, Q3T0X5, Q3T030, Q5E964, P05131, Q2HJ86, Q3ZBU7, Q5E9K0, Q32KL2, Q58DA0, P0CG53, Q3ZBD0, Q3ZCJ7, F1MWE0, Q58DU5, E1B953, Q3ZCA7, F1MLH6, P81948, P81947, Q2KJ46, P23356, Q2KI42, P04896, F1MBI1, P32007, Q3ZBG0, Q0VCX2, F1N461, E1BD83, F2Z4C1, A7MBH9, Q3MHM5, A3KMV5, A4FUZ3, Q5E987, Q3MHN0, F2Z4K0, Q5E946, Q32KN8, P00442, P56701, Q2HJ81, A7MBA2, G5E589, Q2TBX6, F1MXE4, Q2TBP0, E1BQ28, Q3T0Y5, P00517, F1MNF8, Q2YDE4, Q3SZ19 |
| bta05132:Salmonella infection | 60 | 5.11509 | 9.85E-15 | Q3ZCK2, Q2KJD0, Q95122, Q95M18, P61585, G3N132, Q1RMI2, G3MXC8, P62998, F1MX60, Q2HJ86, F1N049, Q3ZBU7, Q76LV2, Q3ZCF0, Q2KJ93, F1MW91, Q3ZCF3, Q3MHR7, Q3ZCJ7, Q3ZBD2, F2Z4F0, E1B953, F1MND1, F1MWN1, P10096, Q148J6, E1BPK6, G3N1U2, G3MXH2, P81948, P63258, P81947, Q09430, F1MCK2, F1MJQ1, F2Z4C1, E1BN47, F1N6J8, Q3T169, Q3MHM5, G3N3N1, F1MIC9, G5E507, F2Z4K0, Q148G7, Q3ZBW5, Q32KN8, F1MNI4, P02584, Q17QH2, A4IF97, Q2HJ81, Q5E9E2, Q58DS9, P84081, P84080, Q58CQ2, Q3T035, A4IFN6, F1MNF8, P04272 |
| bta00010:Glycolysis / Gluconeogenesis | 28 | 2.387042 | 2.12E-14 | F1MJS4, Q5EA79, F1MB08, F1MIM3, Q5E9B1, E1BCW3, Q3SZ62, G3X7N4, E1BNS9, Q0IIG5, Q2KJJ9, F1MX69, A6QLL8, Q1JPA0, Q1JPG7, A6QQ11, Q3ZCJ2, Q3T0P6, Q32KV0, Q2KJE5, Q3ZBD7, Q3T014, Q3SZB7, A5D984, P10096, F1N2L9, E1BME6, F1MZV1, Q5E956 |
| bta04145:Phagosome | 46 | 3.921569 | 6.48E-14 | F1MJQ1, O46563, Q58DR7, Q0VCV6, F2Z4C1, Q3MHM5, Q95122, Q2KJD0, Q2KJD2, A7MAZ2, P61420, Q05204, F2Z4K0, P62998, Q32KN8, Q2HJ86, Q3ZBU7, F1MNI4, O18824, O02659, P31404, P31407, P11019, P21282, Q2HJ81, P31408, A4IFC6, Q3ZCJ7, F1N3A1, Q58DS9, P39942, E1B953, F1MPL6, P23956, A7Z066, F1MNF8, F6RF06, Q3SZJ7, P79251, G3MXJ5, Q3ZBT5, P81948, P63258, P81947, Q2KJ66 |
| bta01200:Carbon metabolism | 34 | 2.898551 | 4.46E-12 | G3MY14, G5E5C8, Q17QI3, F1MB08, F1MIM3, E1BCW3, Q2T9S4, Q3ZCI4, Q9XSG3, Q3SZ62, Q6B855, Q5EAD2, Q3T145, G3X7N4, F6RJ91, Q0IIG5, Q2KJJ9, A7Z014, A6QLL8, F1MBV2, F1MMK2, Q1JPG7, F1N3V0, Q58DK4, Q3T0P6, Q32KV0, Q3ZBD7, Q3SZB7, A5D984, P10096, P33097, A6QR28, E1BME6, F1MZV1, Q5E956, Q2HJ58, Q08E20 |
| bta05014:Amyotrophic lateral sclerosis | 70 | 5.967604 | 6.36E-12 | Q2KIW6, Q5E9F9, F1N6D3, Q3ZCK9, Q3T108, P33672, F1MYH8, Q2KJD0, Q95M59, P00829, G3X757, G3N132, P62194, Q3T0X5, Q3T030, Q5E964, P62998, Q2HJ86, Q3ZBU7, Q5E9K0, Q32KL2, Q58DA0, Q3ZCF0, Q3ZBD1, Q3ZBD0, Q3ZCJ7, Q3ZBD2, F1MWE0, F2Z4F0, Q58DU5, E1B953, F1MUN7, F1MCF5, P81948, P63258, P81947, Q2KJ46, Q09430, Q2KI42, F1MBI1, Q3ZBG0, Q0VCX2, E1BD83, F2Z4C1, Q3MHM5, A4FUZ3, F1MIC9, Q5E987, Q3MHN0, G5E628, F2Z4K0, P37141, Q148G7, Q32KN8, P00442, A5D7T5, Q2HJ23, P56701, P02584, G3N3K4, Q2HJ81, P20072, F1N1A3, A7MBA2, G5E589, Q2TBX6, F1MXE4, Q2TBP0, Q3T0Y5, F1MNF8, P00435, Q2YDE4, P27214, Q3SZ19 |
